# Reference Point and Grid Method-Based Evolutionary Algorithm with Entropy for Many-Objective Optimization Problems

**DOI:** 10.3390/e27050524

**Published:** 2025-05-14

**Authors:** Qi Leng, Bo Shan, Chong Zhou

**Affiliations:** 1 School of Computer, China University of Geosciences, Wuhan 430074, China; qileng@cug.edu.cn; 2 Engineering Research Center of Natural Resource Information Management and Digital Twin Engineering Software, Ministry of Education, Wuhan 430074, China; 3 Hubei Key Laboratory of Intelligent Geo-Information Processing, China University of Geosciences, Wuhan 430074, China; 4 School of Software, Hebei Normal University, Shijiazhuang 050031, China; shanbo@onest.net; 5 School of Information Engineering, Hebei GEO University, Shijiazhuang 050031, China

**Keywords:** many-objective optimization, reference point, grid, entropy

## Abstract

In everyday scenarios, there are many challenges involving multi-objective optimization. As the count of objective functions rises to four or beyond, the problem’s complexity intensifies considerably, often making it challenging for traditional algorithms to arrive at satisfactory solutions. The non-dominated sorting evolutionary reference point-based (NSGA-III) and the grid-based evolutionary algorithms (GrEA) are two prevalent algorithms for many-objective optimization. These two algorithms preserve population diversity by employing reference point and grid mechanisms, respectively. However, they still have limitations when addressing many-objective optimization problems. Due to the uniform distribution of reference points, the reference point-based methods do not obtain good performance on problems with an irregular Pareto front, while grid-based methods do not achieve good results on problems with a regular Pareto front because of the uneven partition of grids. To address the limitations of reference point-based algorithms and grid-based approaches in tackling both regular and irregular problems, a reference point- and grid-based evolutionary algorithm with entropy is proposed for many-objective optimization, denoted as RGEA, which aims to solve both regular and irregular many-objective optimization problems. Entropy is introduced to measure the shape of the Pareto front of a many-objective optimization problem. In RGEA, a parameter α is introduced to determine the interval for calculating the entropy value. By comparing the current entropy value with the maximum entropy value, the reference point-based method or the grid-based method can be determined. In order to verify the performance of the proposed algorithm, a comprehensive experiment was designed on some popular test suites with 3-to-10 objectives. In addition, RGEA was compared against six algorithms without adaptive technology and six algorithms with adaptive technology. A great number of experimental results were obtained showing that RGEA can obtain good results.

## 1. Introduction

Multi-objective optimization problems (MOPs) are ubiquitous in production and life, such as industry and scientific research. The primary objective of MOPs is to identify a set of equilibrium solutions, known as Pareto optimal solutions. When the number of optimization objectives exceeds three, they are termed many-objective optimization problems (MaOPs) [1]. Traditional multi-objective optimization algorithms cannot meet the needs of many-objective optimization. MaOPs pose some new challenges to existing multi-objective evolutionary algorithms [2,3]. First, Pareto dominance becomes degraded for many-objective optimization problems. In the population, there are many non-dominated solutions, which leads to losing selection pressure. Secondly, the recombination operator may be inefficient. Finally, it becomes difficult and expensive to maintain and evaluate the diversity of solutions in the population. Hence, numerous algorithms have been devised for solving these challenges, which can be divided into five categories.

Multi-objective optimization algorithm based on Pareto dominance: On the one hand, this type of algorithm modifies the Pareto dominance domain of the solution, either by expanding or reducing it, thereby enhancing convergence by refining the Pareto dominance mechanism. When handling with many-objective optimization problems, multi-objective optimization algorithms based on Pareto dominance produce a large number of non-dominance solutions in the population, leading to poor pressure of dominance. The representative algorithms are ϵ-dominance [4], grid dominance [5], fuzzy dominance [6], α-dominance [7], etc. On the other hand, for the purpose of ensuring a better selection of the Pareto front, some Pareto dominance-based algorithms have adopted other mechanisms, among which the popular algorithms are the θ dominance-based evolutionary algorithm (θ-DEA) [8], vector angle-based evolutionary algorithm (VaEA) [9], shift-based density estimation algorithm (SDE) [10], knee point-driven evolutionary algorithm (KnEA) [11], etc.

Multi-objective optimization algorithm based on indicators: This kind of algorithms use performance indicators—such as the binary indicators $I_{\varepsilon +}$, R2, and HV—as fitness measures to direct the evolutionary process. The classical algorithms include the indicator-based evolutionary algorithm (IBEA) [12], improved metaheuristic evolutionary multi-objective algorithm based on the R2 indicator (MOMBI-II) [13], and the S metric selection evolutionary multi-objective algorithm (SMS-EMOA) [14]. In order to obtain high-quality solutions, SRA [15] achieves two objectives and mixing based on random bubbling. The indicator-based multi-objective optimization algorithms have good theoretical properties, but they require expensive computation time.

Multi-objective optimization algorithm based on objective dimension reduction: This kind of algorithm forms a minimum conflict objective set by removing redundant objectives so as to simplify the scale of the problem. The widely used algorithms include δ-MOSS [16], NL-MVU-PCA [17], and nonlinear correlation information entropy [18]. According to the similar characteristics of objective dimension reduction and feature selection in machine learning, ref. [19] applied feature selection technology to objective dimension reduction, transforming the objective dimension reduction problem into a multi-objective optimization problem, and they then solved this by an evolutionary algorithm. The algorithm could lose important information when the number of objectives are reduced.

Multi-objective optimization algorithm based on decomposition: This kind of algorithm uses aggregation functions, including the weight sum method (WS) [20], the Tchebycheff method (TCH) [21], and the penalty-based boundary intersection method (PBI) [22], to transform a multi-objective optimization problem into a number of single-objective optimization sub-problems, and it also uses an evolutionary algorithm to optimize these sub-problems at the same time. However, due to the difficulty of maintaining diversity in the traditional decomposition-based multi-objective optimization algorithm (MOEA/D) [23], some improved MOEA/D algorithms have been proposed. The typical algorithms include I-DBEA [24], MOEA/D-DU [25], EFR-RR [25], MOEA/DD [26], MOEA/D-LWS [27], etc.

Multi-objective optimization algorithm based on reference points: This class of algorithm creates a uniformly distributed set of reference points in the objective space to partition it, linking each solution in the population to its nearest reference point. These reference points serve as search directions, guiding the evolutionary process. This class of algorithm shows excellent performance on many-objective optimization problems with a regular Pareto front, but, on the irregular problems, they see a degradation in performance. These sort of algorithms include NSGAIII [2], RVEA [28], crEA [29], etc.

In recent years, multi-objective optimization algorithms that incorporate reference points and grid strategies have garnered significant attention due to their outstanding performance in addressing MaOPs. The representative algorithms are the reference point-based non-dominated sorting evolutionary algorithm (NSGA-III) and the grid-based evolutionary algorithm (GrEA). NSGA-III employs a set of widely distributed reference points to ensure population diversity, and it implements non-dominated sorting. On the other hand, GrEA assesses individuals based on grid domination and grid difference criteria. However, NSGA-III shows excellent performance for regular many-objective optimization problems, but it falls short when handling irregular ones. Conversely, GrEA excels in dealing with irregular many-objective optimization problems but does not perform well with regular ones. In addition, researchers have proposed a large number of methods for dealing with irregular problems; however, the reference point-based method [30,31,32] and the clustering method, or grid-based method [33,34], are still mainly used. There is no reasonable mechanism designed to integrate the reference point-based method and the grid-based method. Hence, a key issue is how to combine the two methods to handle irregular and regular many-objective optimization problems. Entropy has been widely used in the many-objective evolutionary algorithm [35,36,37]. In [38], entropy was treated as a diversity metric to measure the diversity performance of the algorithm. In [39], entropy was researched as a termination criterion for many-objective evolutionary algorithms. However, the entropy was computed based on grid technology. In [40], the entropy was computed based on a reference point and could be viewed as a criterion whether the Pareto front of the many-objective optimization problem was regular or irregular. As such, the entropy mechanism could be employed to combine the two methods. By comparing the current entropy value with the maximum entropy value, the reference point-based method or the grid-based method can be determined.

Drawing from the preceding discussion, this paper introduces a novel algorithm that combines the reference point-based method and the grid-based method for solving regular and irregular many-objective optimization problems (RGEA). RGEA aims to address the shortcomings of NSGA-III and GrEA via a new criterion with entropy, combining the reference point-based method and the grid-based method to improve the diversity of solutions, enhance search efficiency, and ensure the uniform distribution of solutions on the Pareto front. Experiments are conducted on the DTLZ and WFG test problems to verify the effectiveness of RGEA. The proposed algorithm RGEA was compared with six advanced algorithms without adaptive technology, such as MOEA/D, MOEA/D-DE, NSGAIII, GrEA, MSOPS-II, and IBEA, and six advanced algorithms with adaptive technology, such as I-DBEA, ANSGAIII, MOEA/D-AWA, RVEAa, DEAGNG, and AdaW. The primary novel contributions of this paper can be summarized as outlined below.

1. A new algorithm was developed to combine reference point-based and grid-based methods in coping with regular and irregular many-objective optimization problems. The algorithm proposed is to remedy the issue where the reference point-based method has good results in dealing with regular problems but fails to obtain good solutions in dealing with irregular problems, as well as address how the grid-based method has good results in dealing with irregular problems but does not deliver good solutions for regular problems.
2. In this paper, an entropy-based criterion, which combines the reference point-based and grid-based methods, is proposed. In order to determine the interval of the calculated entropy value, we introduced a parameter α. By comparing the current entropy with the maximum entropy, we can determine whether to adopt the reference-point-based approach or the grid-based approach.
3. A comprehensive experimental evaluation was conducted to assess the performance of RGEA. To verify its effectiveness, this paper, using regular and irregular DTLZ1-7 and WFG1-9 test suites for verification, compared RGEA with six evolutionary algorithms without adaptive technology and six evolutionary algorithms with adaptive technology. The large number of experimental results obtained indicate that the proposed algorithm RGEA can achieve good performance on many-objective optimization problems with a regular and irregular Pareto front.

The structure of the subsequent chapters in this paper is organized as follows: Section 2 delves into the definitions of multi-objective optimization problems, differentiating between regular and irregular issues. In addition, it reviews pertinent research concerning reference point-based algorithms and grid-based approaches for many-objective optimization. Section 3 presents the strategy and the overall framework of the proposed algorithm. The experimental setup is detailed in Section 4, and this is followed by Section 5, which discusses the experimental results obtained from various test suites. Lastly, Section 6 provides a concise summary of the paper.

## 2. Preliminaries

### 2.1. Definition of the Multi-Objective Optimization Problem

A MOP can be defined as

(1) $$Minimize\;F\left(x\right)=\left\{f_{1}\left(x\right),\ldots ,f_{m}\left(x\right)\right\}$$

Subjecttox∈D

where *D* denotes the decision space of the problem, and $F:D⟶R_{m}$ is the objective space of a problem, which is composed of m objective functions $f_{1},\ldots ,f_{m}$. When the number of objectives exceeds three, it is named a MaOP [1]. Let $\alpha =\left(\alpha_{1},\alpha_{2},\ldots ,\alpha_{m}\right)$ and $\beta =\left(\beta_{1},\beta_{2},\ldots ,\beta_{m}\right)$, then α is said to dominate β if $\alpha_{i}\leq \beta_{i}$ for all $i=\left(1,\ldots ,m\right)$ and α≠β. A point $x^{*}$ is named Pareto-optimal if there is no x∈D such that $F\left(x\right)$ dominates $F\left(x^{*}\right)$. The set of all the Pareto optimal points is named the Pareto set (PS). $PF=\left\{F\left(x\right)\in R^{m}|x\in PS\right\}$ is named the Pareto front (PF) [39], $z^{*}=\left(z_{1}^{*},\ldots ,z_{m}^{*}\right)$ is named the ideal point if $z_{i}^{*}$ is the minimal value of $f_{i}\left(x\right)$ over the decision space, and $z^{nad}=\left(z_{1}^{nad},\ldots ,z_{m}^{nad}\right)$ is called the nadir point if $z_{i}^{nad}$ is the maximal value of of $f_{i}\left(x\right)$ over the PS.

### 2.2. Definition of Regular and Irregular Problems

A regular multi-objective optimization problem (MOP) is characterized by a Pareto front (PF) that resembles the shape of a simplex. Specifically, when the ideal point of such a problem is considered as the origin, all of the vectors in the positive direction intersect with this ideal point [41]. Conversely, any MOP with PF shapes deviating from this pattern is classified as an irregular MOP. The specific description is to let $\forall v=\left(v_{1},\ldots ,v_{m}\right)$ be a vector, which is subject to $v_{i}\geq 0\;\left(i\in 1,2,\ldots ,m\right)$:

(2) $$Regular\;PF=\left\{PF|∄v=\left(v_{1},\ldots ,v_{m}\right),v\cap PF=\emptyset \right\}$$

(3) $$Irregular\;PF=\left\{PF|\exists v=\left(v_{1},\ldots ,v_{m}\right),v\cap PF=\emptyset \right\}$$

where Formula (2) represents that, for the problem with regular PF, there is no *v* that does not intersect with the PF of the problem, that is, all of the vectors in the positive direction intersect with the PF of the problem. Formula (3) indicates that, for a problem with an irregular PF, there must be a *v* that does not intersect with the PF of the problem.

### 2.3. Reference Point-Based Many-Objective Optimization Algorithms

Moen H. J. F. et al. [42] introduced a novel method for creating a uniformly distributed set of reference points utilizing the Manhattan distance as the basis. This approach involves generating the subsequent generation of solutions by comparing the solutions associated with the same reference points based on this distance metric. K. Deb and Himanshu Jai [2] introduced a concept that combines reference points with a non-dominated sorting. Initially, individuals in the first l−1 layer are chosen through non-dominated sorting. Subsequently, the remaining individuals in the final *l* layer are selected based on two criteria: the distance between the solution and its associated reference point, as well as the number of solutions associated with the same reference point. Yuan Y., Xu H., and Wang B. et al. [8] employed a θ-dominance criterion to allocate solutions to distinct clusters, each represented by specific reference points. Within each cluster, individuals compete against one another. Cheng R. et al. [28] introduced a novel mechanism for partitioning the objective space into multiple subspaces by means of reference vectors. Within each of these subspaces, selection operations are conducted independently. Essentially, this division operation imposes a constraint on the subproblem associated with each reference vector, thereby demonstrating its effectiveness in balancing convergence and diversity within decomposition-based methods [43]. Cai L. et al. [29] suggested utilizing clustering operators to preserve the diversity among solutions. Additionally, they proposed constructing a structured set of reference points to generate a collection of uniformly distributed units within the objective space. Seada H. and Deb K. [44] developed a unified optimization algorithm, which is an extension of NSGAIII, to tackle various multi-objective optimization problems. This algorithm employs a population size larger than the number of reference vectors and incorporates a selection operator. Y. Xiang et al. [45] introduced a novel multi-objective evolutionary algorithm utilizing an adaptive Pareto reference point approach. Within this framework, the choice of reference points is contingent upon the shape of the Pareto optimal front (PF), enabling the calculation of convergence and diversity indices for individuals. W. Yu et al. [46] introduced a decomposition algorithm that leverages an adaptive reference vector Q-learning method. Within this framework, the reinforcement Q-learning framework treats the adaptive process of reference vectors as a reinforcement learning challenge, enabling these vectors to learn from environmental feedback and to select action plans grounded in optimal features. Furthermore, the reference point sampling process utilizes a distributed model to generate novel reference vectors. K. E. Wu et al. [47] introduced a PF tracking approach grounded in B-norm principles. This method involves generating reference points more uniformly across an m-dimensional B-norm surface, which adapts dynamically in response to the true PF being tracked. S. Liu et al. [48] introduced a self-guided reference vector approach, utilizing an enhanced k-means clustering technique to derive reference vectors from the population. Additionally, an angle-based density assessment strategy is employed to dynamically select an adaptive reference vector for guiding the search process. Q. Liu et al. [49] introduced a synchronized adjustment approach for both the reference vector and the aggregation function. Utilizing a local angle threshold, the reference vector is adaptively modified to more closely align with the solution distribution. Furthermore, the weight assigned to the aggregation function is dynamically adjusted based on both the local angle threshold and the age of the reference vector. This coordinated strategy ensures a harmonious balance between diversity and convergence. In the reference point-based approach, the configuration of reference points plays a crucial role in determining the algorithm’s performance. Therefore, the challenge lies in establishing more effective reference points that can efficiently guide the population toward convergence while maintaining diversity, which is essential for the success of this type of algorithm.

### 2.4. Grid-Based Many-Objective Optimization Algorithms

Knowles and Corne [50,51] introduced the PAES procedure, which updates the optimal non-dominated solution set by utilizing a grid crowding mechanism. This mechanism evaluates the crowding degree of each solution based on the number of solutions occupying the same grid location. Consequently, the solution with the highest crowding degree in the archive set is replaced to ensure the generation of an optimal solution suitable for multi-objective problems. Corne et al. [52] introduced the PESA algorithm, which incorporates a novel crowding mechanism. This algorithm divides the normalized space into super-boxes based on an implicitly constructed super-grid. The objective of this division is to combine the convergence capabilities of evolutionary algorithms with the maintenance of population diversity. Laumanns et al. [4] introduced a grid-based method known as ϵ-dominance, representing a novel dominance technique. Deb et al. [53] also created a new method of domination, referred to as ϵ domination. This method divides the objective space into multiple hyperboxes, with each hyperbox containing no more than one individual based on the size of ϵ. Yen and Lu [54] introduced a novel dynamic multi-objective optimization algorithm. The core concept involves assigning each cell in the grid a rank and a density value based on the grid position of the solution and its Pareto dominance relationship. Knowles and Corne [55] proposed new grid-based archiving schemes. As the points in the objective space are generated and archived, the position and size of the grid in the grid space will be adaptively adjusted so that the grid can surround these points. If the archive set reaches the capacity limit, the grid-based method can well select the points removed from the archive. Rachmawati et al. [56] developed a dynamic method for adjusting the grid, which identifies and adjusts the grid size according to the average occupancy and neighbor occupancy. Y. Zhou et al. [57] developed the ‘box’ function, where the objective space is divided into multiple grids based on the ‘box’ function and a vector is returned by the ‘box’ function to represent a grid vertex. The ‘update’ and ‘box’ functions that are adopted ensure that each grid contains at most one solution. A. G. Hern et al. [58] developed an improved ϵ-dominance algorithm, which overcomes the problem that some non-dominated solutions are lost from the hyper-grid used in the archive due to the way of selecting solutions in each box in the original method. Karahan and Koksalan [59] introduced a multi-objective optimization algorithm centered on the concept of territory. The core idea involves dividing the objective space into multiple hyperboxes, all of which are of equal size. To prevent overcrowding, each hyperbox is designed to contain at most one individual, and a territory is defined around each individual to maintain high diversity. This approach bears some resemblance to the method proposed in [53]. X. Cai et al. [60] introduced an inverted generational distance metric based on a grid to assess both the convergence and diversity of the population. X. He et al. [61] introduced a method for balancing convergence and diversity through a combination of decomposition-based and Pareto dominance selection based on grids. M. He et al. [62] introduce an evolutionary algorithm leveraging non-uniform grids. Within this system, a dynamic non-uniform meshing approach and an offset-driven density assessment technique are employed to differentiate non-dominated solutions within grid coordinates. V. Palakonda et al. [63] introduced a hybrid approach that partitions the objective space through clustering-based mating selection, utilizing Euclidean distance to identify potential offspring solutions. Subsequently, combined with grid-based one-to-one environment selection, grid coordinate point distance is employed to select the most promising solution. While the grid-based method offers a uniform partition, it tends to result in wasted computing resources, and the algorithm’s performance is significantly affected by the size of the grid.

## 3. The Proposed Algorithm

### 3.1. The Main Idea of the Proposed Algorithm

In this paper, we introduce an evolutionary algorithm, specifically designed for solving many-objective optimization problems, called the Reference Point and Grid Method-based Evolutionary Algorithm (RGEA). RGEA combines the benefits of a diversity evaluation method based on reference points, which aids in maintaining population diversity and finding high-quality solutions on the regular Pareto front, with the advantages of introducing grid dominance and grid difference to accelerate the convergence on the irregular Pareto front when evaluating individuals using a grid-based strategy. The ultimate aim is to enhance the algorithm’s capacity to address many-objective optimization problems. RGEA primarily consists of the generation of reference points, normalization, entropy calculation, and grid division. Since entropy serves as a measure to quantify the diversity of solutions in the population for many-objective optimization problems, we incorporate entropy into the algorithm to determine whether to adopt the reference point-based approach or the grid-based approach. In order to calculate the entropy value at each fixed iteration, we introduce a parameter α to control the interval for calculating the entropy value. By comparing the current entropy value with the maximum entropy value, the search approach based on the reference point or the grid can be determined, thereby ensuring both great convergence and diversity. The structure of the RGEA is outlined below.

The primary concept of Algorithm 1 involves introducing a parameter to regulate the number of iterations, which is used to determine the interval for calculating the entropy value. By comparing the current entropy with the maximum entropy, either the reference point-based approach or the grid-based method is adopted. The union operation is employed to combine the parent population with the offspring population, thereby generating a new population, as shown in Step 3. Then, the new population is non-dominated sorted to select the next population, as shown in Step 4. The population $S_{t}$ consists of $\left(F_{1},F_{2},\ldots ,F_{l}\right)$, where *l* represents the last layer of the selected non-dominated front. Subsequently, the adaptive normalization process is performed. If the kind is equal to 1, then K individuals are selected from the last non-dominated layer by the niche retention technique, as shown in Steps 14–18. Otherwise, K individuals are chosen from the final non-dominated layer by means of the grid-based method, as shown in Steps 19–22. A parameter α is introduced for the purpose of controlling the number of iterations, which, in turn, determines the interval for calculating the entropy value. The current entropy and the maximum entropy are compared to determine whether the reference point-based approach or the grid-based approach is adopted. When the current entropy reaches the maximum entropy, the grid-based method is used, as shown in Steps 24–27. The key technologies of RGEA are described in the following sections.
**Algorithm 1:** Framework of RGEA**Input:**    *H* structured reference ponits $Z^{s}$ or supplied aspiration ponits $Z^{a}$    *P*: population    *N*: population size    maxGen: maximum number of iterations    *N* uniformly distributed weight vectors: $\lambda^{1},\ldots ,\lambda^{N}$    div: the size of grid division    α: the interval for calculating the entropy value**Initialization:**     *t* = 1     $P_{t}$ = Initialize(*P*)     Compute maximum entropy: *MaxEntropy*;**Optimization:**    while t < maxGen do    1: $S_{t}=\emptyset$, i=1,    2: $Q_{t}$ = Genetic Recombination and Mutation($P_{t}$)    3: $R_{t}=P_{t}⋃Q_{t}$    4: $\left(F_{1},F_{2},\ldots \right)$ = Non-dominated Sorting$\left(R_{t}\right)$    5: while $\left|S_{t}\right|<N$ do    6:   $S_{t}=S_{t}⋃F_{i}$    7:   i=i+1    8: Final Front to Incorporate: $F_{l}=F_{i}$    9: if $\left|S_{t}\right|=N$ then  10:   $P_{t+1}=S_{t}$  11: else  12:  $P_{t+1}={⋃}_{j=1}^{l-1}F_{j}$  13:  Points Selected From $F_{l}:K=N-\left|P_{t+1}\right|$  14: if kind = 1  15:   Normalize Objectives and Establish Reference Set $Z^{r}$:$normalize\left(f^{n},S_{t},Z^{r},Z^{s},Z^{a}\right)$  16:   Associate each member of with a reference point: $\left[\pi \left(s\right),d\left(s\right)\right]=Associate\left(S_{t},Z^{r}\right)$  17:   Compute niche count of reference point $j\in Z^{r}:\rho_{j}=\sum_{s\in S_{t}/F_{l}}\left(\left(\pi \left(s\right)=j\right)?1:0\right)$  18:   Choose members one at a time from $F_{l}$ to construct         $P_{t+1}$:$Niching\left(K,\rho_{j},\pi ,d,Z^{r},F_{l},P_{t+1}\right)$  19: else  20:   $Grid_{-}setting\left(S_{t}\right)$  21:   $Fitness_{-}assignment\left(S_{t}\right)$  22:   $P_{t+1}=Environmental_{-}selection\left(S_{t}\right)$  23: end if  24: if t%α==0  25:   Calculate CurrentEntropy: CurrentEntropy  26:   if MaxEntropy - CurrentEntropy <= 0, then  27:     kind = 2  28: end if  29: end if  30: end if  31: t=t+1  32: end**Output:**   *P*

### 3.2. Generation of Reference Points

By selecting the reference point sets in the objective space, the algorithm benefits from improved stratification and more effective solution selection through the choice of a reference point. The role of the reference point is akin to stratified sampling in statistics, which covers the whole objective space by uniformly distributed reference points, thereby guaranteeing the variety and even dissemination of solutions. When selecting a reference point, three essential criteria must generally be met: 1. Reference points must be evenly distributed within the objective space to ensure comprehensive coverage of the entire space. 2. The number of reference points should be moderate. 3. Having too many reference points can lead to reduced search efficiency. In this paper, the reference point set is generated using the boundary intersection method of DaS and Dennis to construct a fully intelligent approach [22]. This method can generate a uniformly distributed reference point set in the objective space, with a predefined integer representing the division on each coordinate axis. In this approach, reference points are obtained by sampling from a unit, and the quantity of these reference points (denoted as *H*) can be determined using the following calculation method:

(4) H=p+m−1p.

**Figure 1:**
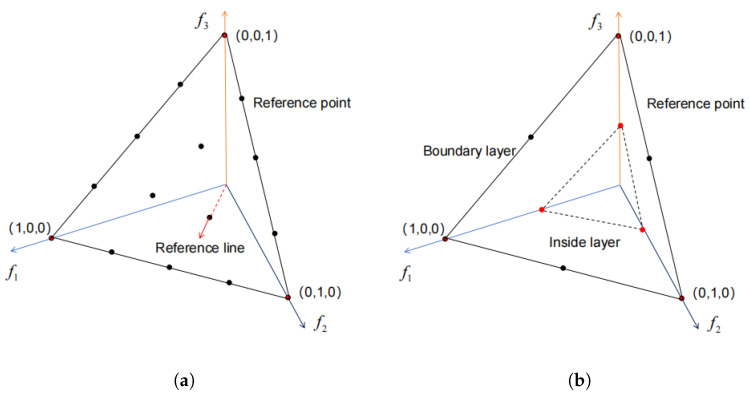
Illustration of the arrangement of reference points in a three-objective space: (**a**) Illustration of the arrangement of a single layer of reference points in three-objective space, and (**b**) illustration of the arrangement of reference points for two layers in three objective spaces.

It can be inferred from Equation (4) that the quantity of reference points is affected by both the dimension *M* and the integer *p* associated with the objective. When dealing with a problem involving three objectives and assigning *p* the value of 4, the total count of reference points becomes *H* = 15. For a clear explanation, Figure 1a depicts the arrangement of reference points for a single layer on a three-objective space. When *p* equals *M*, the number of reference points is considered adequate. However, in the context of a many-objective space, even with *p* set to *M*, there is a steep increase in the number of reference points, significantly exacerbating the computational time complexity. For instance, when *M* is set to 6, then *p* is equal to 6, which will necessitate the use of 462 reference points. Moreover, if only p is reduced, then this will lead to a sparse distribution of reference points and a loss of diversity among them. To address this issue, the literature [2] has introduced a method of distributing reference points in two layers, utilizing a relatively small *p* value for the inner points. Assuming that $p_{1}$ and $p_{2}$ represent the outer partition and the inner partition, respectively, then the overall count of reference points (denoted as *H*) is calculated as follows:

(5) H=p1+M−1p1+p2+M−1p2.

Figure 1b illustrates the arrangement of reference points for two layers in three objective spaces, with the outer layer delineated by $p_{1}$ = 2 and the inner layer delineated by $p_{2}$ = 3.

### 3.3. Grid-Based Mechanism

**Figure 2:**
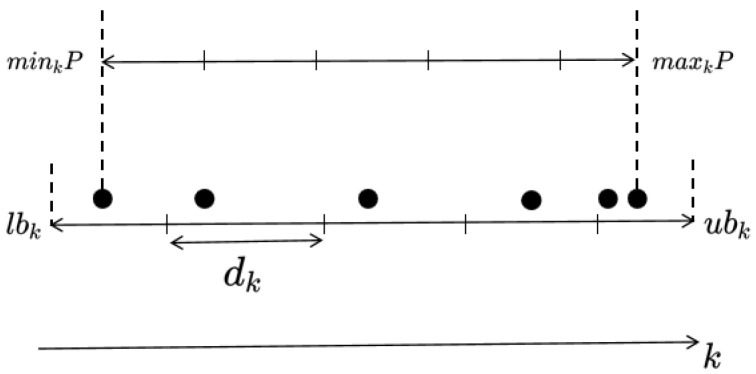
Configuration of the grid for the *k*th objective.

The concept of a grid-based methodology involves dividing the objective space into numerous grids and, subsequently, utilizing these grids to pinpoint the location of an individual in the objective space. In this paper, the adaptive grid construction method [51] was employed to dynamically adjust the size and position of the grids based on the evolving population. Figure 2 shows the grid settings in the *k*-th objective.

As depicted in Figure 2, $min_{k}\left(P\right)$ and $max_{k}\left(P\right)$ denote the lowest and highest values of the *k*-th objective within population P, respectively. According to Formulas (6) and (7), the lower and upper boundaries for the *k*-th objective are established.

(6) $$lb_{k}=min_{k}\left(P\right)-\left(max_{k}\left(P\right)-min_{k}\left(P\right)\right)/\left(2\times div\right),$$

(7) $$ub_{k}=max_{k}\left(P\right)+\left(max_{k}\left(P\right)-min_{k}\left(P\right)\right)/\left(2\times div\right),$$

where div signifies the number of divisions in the objective space along each dimension. The lower and upper limits of the mesh in the *k*-th objective are denoted by $lb_{k}$ and $ub_{k}$, respectively. For instance, in Figure 2, div is equal to 5. Consequently, the objective space is partitioned into $div^{M}$ hypercubes. Therefore, the width $d_{k}$ of the hypercube in the first objective can be calculated using Formula (8).

(8) $$d_{k}=\left(ub_{k}-lb_{k}\right)/div.$$

Therefore, the individual’s grid position in the *k*-th objective can be ascertained based on $lb_{k}$ and the corresponding width $d_{k}$. The specific calculation formula is shown in (9):

(9) $$G_{k}\left(x\right)=\left\lfloor \left(F_{k}\left(x\right)-lb_{k}\right)/d_{k}\right\rfloor .$$

**Figure 3:**
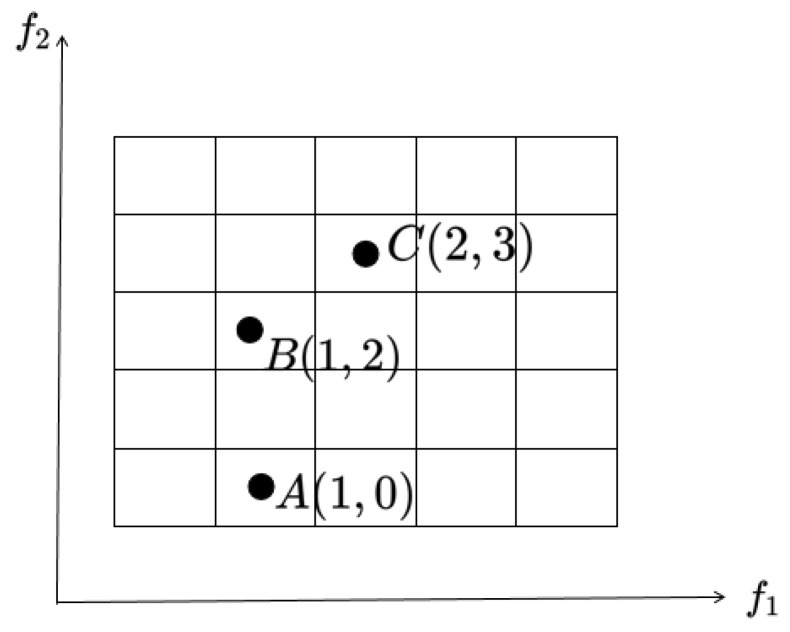
A depiction of individuals positioned within a grid in a bi-objective space.

In Formula (9), $\left\lfloor \cdot \right\rfloor$ denotes the downward integer function, $G_{k}\left(x\right)$ stands for the grid coordinates of individual *x* in the *k*-th objective, while $F_{k}\left(x\right)$ signifies the actual objective value of individual *x* in the *k*-th objective. The width of the hypercube in the *k*-th objective is denoted by $d_{k}$. As illustrated in Figure 2, the grid coordinates of the individuals from left to right in the *k*-th objective are 0, 1, 2, 3, and 4, respectively. Taking the bi-objective space in Figure 3 as an example, the grid coordinates of individuals A, B, and C are (1, 0), (1, 2), and (2, 3), respectively.

After determining the grid coordinates of each individual, the grid difference $\left(GD\right)$ between the individuals can be determined. The exact calculation formula is shown in (10):

(10) $$GD\left(x,y\right)=\sum_{k=1}^{M}\left|G_{k}\left(x\right)-G_{k}\left(y\right)\right|.$$

In this context, $G_{k}\left(x\right)$ and $G_{k}\left(y\right)$ denote the grid coordinates of individuals *x* and *y* in the *k*-th objective, respectively, where *M* represents the dimensionality of the objective space. It is evident that the grid difference is greatly influenced by the partition count, div, and the range of the grid difference extends from 0 to $M\left(div-1\right)$. An increase in the number of partitions, div, leads to a decrease in the size of each grid cell, which, in turn, results in a larger grid difference.

To facilitate the population’s evolution toward the Pareto front and ensure an even distribution along the PF, the fitness assessment of individuals must encompass two key indicators: convergence and diversity. In this paper, a grid-based approach, utilizing three techniques to assign fitness values to individuals, was employed: grid sorting $\left(GR\right)$, grid crowding distance $\left(GCD\right)$, and grid coordinate point distance $\left(GCPD\right)$.

GR reflects the population’s convergence by ranking individuals based on their grid coordinates across each objective. Specifically, for each individual, $\left(GR\right)$ is calculated as the aggregate of its grid coordinates across all objectives. The exact calculation formula is shown in (11):

(11) $$GR\left(x\right)=\sum_{k=1}^{M}G_{k}\left(x\right).$$

In this context, $G_{k}\left(x\right)$ signifies the grid coordinates of individual *x* for the *k*-th objective, and *M* denotes the total number of objectives. Estimating the density of solutions plays a crucial role in allocating fitness, as a well-distributed set of solutions can direct the search toward the entire Pareto front. Consequently, a density estimator for individual *x*, termed as GCD, is introduced. The precise Formula (12) for GCD is outlined as follows:

(12) $$GCD\left(x\right)=\sum_{y\in N\left(x\right)}\left(M-GD\left(x,y\right)\right).$$

In this context, $GD\left(x,y\right)$ signifies the grid difference between individual *x* and individual *y*, while $N\left(x\right)$ denotes the set of neighbors for individual *x*. An individual *y* is considered a neighbor of individual *x* if $GD\left(x,y\right)$ is less than *M*. The farther the neighbor position is, the smaller the contribution to it is.

For convergence and diversity metrics, even if GR and GCD provide a good reference for the population, there will be cases where individuals cannot be distinguished because they are calculated according to the individual’s grid coordinates and they have an integer value, which indicates that the individual’s GR and GCD values may be the same. Here, inspired by the ϵ-MOEA [49] algorithm, this paper calculated the normalized Euclidean distance between an individual and its corresponding Utopian point within the hypercube, which represents the optimal corner point of that individual’s hypercube and is named GCPD. The precise Formula (13) for GCPD is outlined as follows:

(13) $$GCPD\left(x\right)=\sqrt{\sum_{k=1}^{M}{\left(\left(F_{k}\left(x\right)-\left(lb_{k}+G_{k}\left(x\right)\times d_{k}\right)\right)/d_{k}\right)}^{2}}$$

In this context, $G_{k}\left(x\right)$ represents the grid coordinates of individual *x* for the *k*-th objective, $F_{k}\left(x\right)$ stands for the objective value of individual *x* on the *k*-th objective, $lb_{k}$ denotes the lower boundary of the grid in the *k*-th objective, and $d_{k}$ signifies the width of the hypercube in the *k*-th objective. From Formula (13), it is evident that a lower GCPD is more favorable.

**Figure 4:**
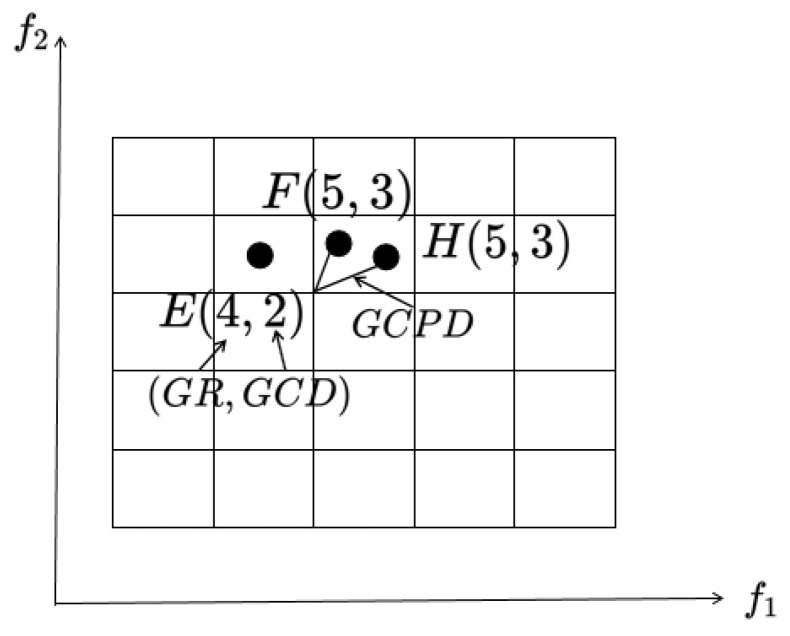
A graphic representation of the fitness allocation of an individual. The numbers in brackets denote GR and GCD, respectively.

Based on the analysis of the above three indicators GR, GCD, and GCPD, which are used to evaluate the status of the population, it can be seen that they can gradually effectively distinguish individuals. The designated diagram is illustrated in Figure 4.

The fitness assignment based on a grid is mainly conducted to search the optimal solution in the considered frontier. The specific method is to pass the above three criteria. Among all the criteria, the lower value is more desirable. The specific working principle refers to Algorithm 2:
**Algorithm 2:** $Search\;optimal\left(P\right)$**Input:**    $p_{opt}$: optimal solution in *P*    $p_{i}$: the *i*-th solution *P***Optimization:**    1: $p_{opt}⟵p_{1}$    2: for i=2 to $\left|P\right|$ do    3:   if $GR\left(p_{i}\right)<GR\left(p_{opt}\right)$ then    4:     $p_{opt}⟵p_{i}$    5:   else if $GR\left(p_{i}\right)=GR\left(p_{opt}\right)$ then    6:     if $GCD\left(p_{i}\right)<GCD\left(p_{opt}\right)$ then    7:       $p_{opt}⟵p_{i}$    8:     else if $GCD\left(p_{i}\right)=GCD\left(p_{opt}\right)$ then    9:       if $GCPD\left(p_{i}\right)<GCPD\left(p_{opt}\right)$ then    10:          $p_{opt}⟵p_{i}$    11:       end if    12:     end if    13:   end if    14: end for**Output:**         $p_{opt}$

### 3.4. Entropy Calculation

Entropy, a fundamental concept introduced by Claude Shannon in 1948 in the framework of information theory, serves as a metric to assess the degree of uncertainty or lack of clarity in information. Specifically, in the realm of information theory, the entropy $H\left(X\right)$ of a discrete random variable *X* is defined as follows:

(14) $$H\left(X\right)=-\sum_{i=1}^{n}p\left(x_{i}\right)\ln\left(p\left(x_{i}\right)\right)$$

where $p\left(x_{i}\right)$ represents the probability that the random variable *X* takes the value $x_{i}$, and *N* denotes the total number of possible values that *X* can take. Considering the scenario presented in this paper, *N* solutions are allocated to *K* reference vectors, such that each reference vector is associated with $n_{1},n_{2},\ldots n_{k}\left(n=n_{1}+n_{2}+\ldots +n_{k}\right)$ solutions. Furthermore, the probability of a solution being linked to the *i*-th reference vector is denoted as $p_{i}$.

(15) $$H\left(X\right)=-\sum_{i=1}^{N}p_{i}\ln\left(p_{i}\right)=-\sum_{i=1}^{N}\frac{1}{N}\ln\frac{1}{N}=\sum_{i=1}^{N}\frac{1}{N}\ln N=\ln N$$

where, if $p_{i}=0$, then $p_{i}\ln\left(p_{i}\right)=0$. When the probability distribution is uniformly distributed, that is $p_{i}=1/N$ for all *i*, then the entropy reaches its maximum value, $H_{max}=\ln\left(N\right)$. A higher entropy value indicates a greater degree of information uncertainty and a larger amount of information. In the realm of optimization problems, entropy holds significant applied value. By incorporating the concept of entropy, we can more accurately describe the complexity and uncertainty of the problem, thereby guiding the design and implementation of optimization algorithms. Specifically, in multi-objective optimization problems, entropy serves as a useful metric for assessing the diversity and uniformity of solutions. These problems typically involve multiple objective functions, which may exhibit conflicts and trade-offs. By calculating the entropy of the solutions, we can effectively gauge their diversity and uniformity, thus informing the selection and refinement of the algorithm. The framework for calculating entropy is outlined below.

Algorithm 3 focuses on two main processes: the association operation and entropy calculation. The association operation aims to link each solution in the population with a specific reference point. This is achieved by evaluating the vertical distance between the objective value of each solution and a reference line and then grouping solutions associated with the same reference line into clusters. The entropy calculation, on the other hand, serves to determine the probability distribution of the population, facilitate the selection of an appropriate decomposition algorithm, and enhance population diversity. The association operation involves three key steps, as shown in Steps 1–3: 1. Calculating the distance of each solution to each reference vector. 2. Associating each solution with its nearest reference point. 3. Counting the number of solutions associated with each reference point. Regarding entropy calculation, the process begins with initializing the entropy value to 0, as shown in Step 4. Subsequently, the entropy is computed based on the probability distribution of the solutions, spanning from Step 5 to Step 12.
**Algorithm 3:** Calculate the Entropy**Input:**     *N*: The population size     *N* uniformly distributed weight vectors: $\lambda^{1},\ldots \lambda^{N}$     rho: the count of solutions associated with each reference point**Association:**    1: Measure the distance between each solution and each reference vector: *Cosine*    2: Match each solution with its closest reference point: *pi*    3: Compute the count of solutions associated with each reference point: *rho***Calculate the Entropy:**    4: entropy = 0    5: i = 1    6: while i≤N do    7: if $Num\left(i\right)\neq 0$, then $p=Num\left(i\right)/N$, entropy = entropy +$p*\log\left(p\right)$    8: end    9: if the loop ends    10:  entropy = −entropy    11: end if    12: i = i + 1    13: end**Output:**     entropy

### 3.5. Analysis of Computational Complexity

The computational complexity of RGEA is evaluated over a single iteration. Algorithm 1 primarily encompasses three components: non-dominated sorting, environment selection based on either reference points or the grid, and entropy calculation. The non-dominated sorting step has a complexity of $O\left(mN^{2}\right)$, where *m* represents the number of objectives and *N* denotes the population size. Both the reference point-based and grid-based environment selection steps have a complexity of $O\left(N^{2}\right)$. Additionally, the entropy calculation requires $O\left(N\right)$ computations. On the whole, the overall complexity of RGEA is $O\left(mN^{2}\right)$.

## 4. Experiment Design

### 4.1. Experiment Settings

The specific experimental configurations are outlined below.

(1) Characteristics of the test problems: For the test problems involving 3, 5, 8, and 10 objectives, the population sizes are set to 91, 210, 156, and 275, respectively. Each algorithm is independently run 20 times on each benchmark. The algorithms considered all have an equal number of evaluations for the same test problem, and the maximum number of fitness evaluations is detailed in Table 1. Additionally, the stopping criterion for these runs is not specified here, but it is presumably based on reaching the maximum number of evaluations listed in Table 1.
(2) Configuration of genetic operators: In this paper, our primary focus is on the simulated binary crossover operator and the polynomial mutation operator. We set the crossover probability to 1.0 and the mutation probability to 1/n, which represents the total number of decision variables. Additionally, the distribution index for both the crossover and mutation operators is set at 20.
(3) Parameters in MOEA/D and GrEA: For MOEA/D, the neighborhood size is determined by rounding 10% of the population size to the nearest integer for the given benchmark problem. In the case of GrEA, the grid division size follows the guidelines outlined in reference [5].
(4) Computation of performance indicators: In this paper, the performance of each algorithm was assessed using the inverted generational distance (IGD) indicator and the hypervolume (HV) indicator, of which the specific calculation reference is in Section 4.4. The average value of these two index values in 20 runs was calculated on each test problem. Another important step in evolutionary computation is to normalize the objective space to a unified scale. The primary approach involves normalizing the objective space to the interval ${\left[0,1\right]}^{m}$ based on the minimum and maximum values of each objective, as derived from the true Pareto front of each benchmark problem. Consequently, the normalized ideal point shifts to a zero vector, while the nadir point transforms into (1, 1, …, 1).

**Table 1:** Characteristics of the test problems.

Test Problems	Objectives (M)	Variables (D)	Evaluations	Pareto Front
DTLZ1	3, 5, 8, 10	M-1 + 5	50,000	Regular
DTLZ2, DTLZ4	3, 5, 8, 10	M-1 + 10	20,000	Regular
DTLZ3	3, 5, 8, 10	M-1 + 10	50,000	Regular
DTLZ5, DTLZ6	3, 5, 8, 10	M-1 + 10	20,000	Irregular
DTLZ7	3, 5, 8, 10	M-1 + 20	20,000	Irregular
WFG1-3	3, 5, 8, 10	24	N∗500	Irregular
WFG4-9	3, 5, 8, 10	24	N∗200	Regular

### 4.2. Benchmark

In this paper, two popular continuous extensible multi-objective test problem sets DTLZ [64] and WFG [65] are used. To evaluate the efficacy of RGEA and other comparative algorithms in handling both regular and irregular challenges, this paper utilizes DTLZ1-7 and WFG1-9 test problems, which involve objectives ranging from 3 to 10. Specifically, DTLZ1 satisfies the condition A = E for its Pareto front (PF), while the remaining DTLZ problems adhere to the GFD criterion. For the DTLZ problems, the number of decision variables (n) is determined by the formula n = m + r − 1, where r is set to 5 for DTLZ1 and 10 for the other DTLZ2-6 problems, while r is set to 20 for the DTLZ7 problem. As for the WFG test problems, based on reference [2], the number of decision variables is fixed at n = 24. Additionally, the location-dependent variable k is set to m − 1, and the distance-dependent variable l is set to n − k.

### 4.3. Algorithms in Comparison

To evaluate the effectiveness of RGEA, this paper considered ten advanced EMO algorithms as comparison algorithms. The implementation of all algorithms was carried out within the PlatEMO framework [66]. The comparison algorithm is introduced as follows.

NSGAIII [2]: By establishing a uniformly distributed reference point beforehand to direct the algorithm’s search, the resulting Pareto front can be made to be as evenly spread as possible while preserving the non-dominated individuals situated close to this reference point.

GrEA [5]: The algorithm utilizes grid division to partition the solution space into multiple grid units, effectively narrowing the search scope and bolstering the search efficiency. These grid units possess inherent attributes that encapsulate both convergence and diversity information. Each individual within the grid occupies a specific position and, by comparing the grid position of an individual with those of others, one can assess its convergence. Furthermore, by counting the number of solutions sharing the same or similar grid positions, the diversity of an individual can be evaluated.

MOEA/D [23]: The algorithm employs the divide-and-conquer approach to transform a multi-objective problem (MOP) into several scalar quantum problems, with each sub-problem formulated by incorporating a set of uniformly distributed weight vectors. The core process involves updating the solutions in proximity to each sub-problem through the use of an aggregation function. This type of algorithm has demonstrated its efficacy in both multi-objective optimization [67] and many-objective optimization scenarios [68].

MOEA/D-DE [67]: This algorithm introduces a novel mechanism, i.e., it modifies the traditional concepts of SBX [69] and the polynomial mutation operator [69]; instead, it proposes the use of the differential evolution (DE) [70] operator for generating offspring. To ensure diversity is maintained, two supplementary measures are implemented.

I-DBEA [24]: This algorithm employs systematic sampling to produce uniformly distributed reference points and connects each solution to a reference point through a straightforward preemptive distance comparison method. To strike a balance between convergence and diversity, two separate distance metrics are utilized.

MSOPSII [71]: This algorithm represents an enhanced version of the MSOPS algorithm. It eliminates the need for initial prior designer intervention by utilizing automatic objective vector generation. Additionally, it simplifies analysis and performs more intricate constraint processing through the adoption of a novel fitness assignment approach.

IBEA [12]: This algorithm employs binary tournaments for mating selection. During the environmental selection process, the objective is to eliminate the weakest individual from the population and iteratively update the fitness values of the remaining solutions.

MOEADAWA [72]: This algorithm builds upon the TCH decomposition method utilized in MOEA/D, introducing a novel concept, i.e., a weight vector initialization method alongside the adaptive allocation of sub-problem weight vectors. This approach aims to achieve greater uniformity within the population.

ANSGAIII [73]: This algorithm incorporates adaptive reference points and a non-dominated sorting technique. The core concept revolves around utilizing adaptive reference points to maintain population diversity and mitigate premature convergence to local optimal solutions.

RVEAa [28]: This algorithm is an enhanced version of RVEA that employs a novel adaptive strategy to dynamically adjust the distribution of reference vectors in accordance with the scale of the objective function.

DEAGNG [74]: This algorithm employs a growing neural gas network to acquire knowledge of the topological structure inherent in the PF. Subsequent adjustments are made to both the reference vectors and the scalarizing functions guided by the topological neighbor relationships that have been elucidated through the learning process.

AdaW [75]: This algorithm initially preserves an archive set containing well-distributed, non-dominated solutions. Subsequently, it constructs reference vectors in unexplored yet promising regions based on the solution distribution within the archive set, while eliminating those located in overcrowded or less favorable areas.

### 4.4. Selection of Evaluation Metrics

To evaluate and compare the performance of RGEA against the comparison algorithms, this experiment selected the widely used comprehensive performance metrics that simultaneously evaluate convergence and diversity: inverted generational distance (IGD) [76] and hypervolume (HV) [77].

The IGD indicator serves as a metric to quantify the distance between the computed Pareto-approximate solution set and a predefined reference set. Specifically, the reference set comprises points that are uniformly distributed along the Pareto front (PF), whereas the solution set consists of points approximating the PF. Subsequently, the IGD value for the solution set is characterized as follows:

(16) $$IGD\left(P^{*},A\right)=\frac{\sum_{v\in P^{*}}d\left(v,A\right)}{\left|P^{*}\right|}.$$

In this context, $d\left(v,A\right)$ denotes the Euclidean distance in the objective space between solution *v* and solution set *A*. Provided that $\left|P^{*}\right|$ is sufficiently large to adequately represent the Pareto front, $IGD\left(P^{*},A\right)$ can serve as a comprehensive metric for evaluating both the convergence and diversity of the solution set. This is performed because, to achieve a lower $IGD\left(P^{*},A\right)$ value, solution set *A* must be sufficiently close to the Pareto front in the objective space, thereby ensuring that every portion of $P^{*}$ has a corresponding solution in *A* for adequate representation.

Hypervolume (HV): Consider *A* as the approximate solution set representing the Pareto front, and let $r={\left(r_{1},r_{2},\ldots ,r_{m}\right)}^{T}$ be a reference point in the objective space. This reference point *r* is dominated by all the objective vectors within the solution set *A*. Subsequently, the hypervolume (HV) associated with the reference point *r* quantifies the volume of the objective space that is dominated by the solution set *A* and is bounded by the reference point *r*. The precise Formula (17) for HV is outlined as follows:

(17) $$HV\left(A,r\right)=\delta \left(\underset{f\in A}{⋃}\left[f_{1}\left(x\right),r_{1}\right]\times \cdots \times \left[f_{m}\left(x\right),r_{m}\right]\right).$$

In this context, δ represents the Lebesgue measure, and it is utilized for measuring the volume. Furthermore, $\left[f_{m}\left(x\right),r_{m}\right]$ denotes the hypervolume formed by the reference point $r_{m}$ and the *m*-th solution in the solution set. The choice of the reference point influences the precision of the hypervolume indicator value to a certain degree. A higher HV indicates a more superior non-dominated solution set.

### 4.5. Nonparametric Statistics Analysis

In this paper, the Wilcoxon signed rank test [78,79] was employed with a significance level set at 5% to demonstrate the statistical significance between the RGEA and ten other algorithms across 20 independent runs. The table highlights the best average results for IGD and HV, which were calculated by each algorithm on each test problem with a blue background. The experimental results utilized three symbols, “+”, “−”, and “=”, to indicate, respectively, when the comparison algorithms outperformed RGEA, when RGEA performed more excellently, and when there was no significant difference between RGEA and the other comparison algorithms.

## 5. Experimental Results and Analysis

### 5.1. Comparison with Six Evolutionary Algorithms Without Adaptive Technology

This section shows the IGD values of RGEA and six evolution algorithms without adaptive technology, such as MOEA/D, MOEA/D-DE, NSGAIII, GrEA, MSOPSII, and IBEA, on the DTLZ1-7 and WFG1-9 test problems. The experimental results are shown in Table 2. RGEA performs better than the other six algorithms on all test problems. In the analysis of the IGD metric, RGEA performed best on the DTLZ1 problem with 3 objectives, the DTLZ3 problem with 3 objectives, the DTLZ5 problem with 3 objectives, the WFG2 problem with 3 objectives, the WFG4 problem with 3 and 5 objectives, the WFG6 problem with 3 and 5 objectives, the WFG7 problem with 5 objectives, the WFG8 problem with 3 objectives, and the WFG9 problem with 3 objectives.

When RGEA was compared with MOEA/D, for the regular DTLZ1-4 problems, RGEA performed better on the DTLZ4 problem with 5, 8, and 10 objectives. For the irregular DTLZ5-7 problems, RGEA performed better on the DTLZ5 and DTLZ6 problems with 3 objectives, and it also performed better on the DTLZ7 problem with 3, 5, and 8 objectives. For the WFG1-9 problem with 3-to-10 objectives, RGEA performed better than MOEA/D.

When RGEA is compared MOEA/D-DE, for the regular DTLZ1-4 problems, RGEA performs better on the DTLZ1 and DTLZ3 problems with 3 and 5 objectives, and performs better on the DTLZ4 problem with 5, 8, and 10 objectives. For the DTLZ2 problem with 3-to-10 objectives, RGEA performs better than MOEA/D-DE. For the irregular DTLZ5-7 problems, RGEA performs better on the DTLZ5 problem with three objectives, and performs better on the DTLZ7 problem with three or five objectives. For the WFG1-9 problem with 3-to-10 objectives, RGEA performs better than MOEA/D-DE.

When RGEA was compared with NSGAIII, RGEA performed better on the DTLZ1 problem with 3 objectives and the DTLZ2 and DTLZ3 problems with 8 objectives (with respect to the regular DTLZ1-4 problem). Regarding the irregular DTLZ5-7 problems, RGEA performed better on DTLZ5-7 problems with 3-to-10 objectives. For the irregular WFG1-3 problems, RGEA performed better on the WFG1-3 problems with 3-to-10 objectives. For the regular WFG4-9 problems, RGEA performed better on the WFG8 problem with 8 objectives and the WFG9 problem with 3 objectives.

When RGEA was compared with GrEA, RGEA performed better on the DTLZ1 problem with 3, 8, and 10 objectives; the DTLZ2-3 problems with 3, 5, and 8 objectives; and the DTLZ4 problem with 5 objectives (in terms of the regular DTLZ1-4 problems). Concerning the irregular DTLZ5-7 problems, RGEA performed better on the DTLZ5 problem with 3, 8, and 10 objectives and the DTLZ6 problem with 3 objectives. Regarding the irregular WFG1-3 problems, RGEA performed better on the WFG1 problem with 8 objectives, the WFG2 problem with 3 and 5 objectives, and the WFG3 problem with 10 objectives. For regular WFG4-9 problems, RGEA performed better on the WFG4 and WFG9 problems with 3 objectives; the WFG5-7 problems with 3 and 5 objectives; the WFG8 problems with 3, 5, and 10 objectives; and the WFG9 problem with 3 objectives.

When RGEA was compared with MSOPSII, RGEA performed better on the DTLZ1 and DTLZ3 problems with 3 objectives; the DTLZ2 problems with 3, 5, and 8 objectives; and the DTLZ4 problems with 3-to-10 objectives according to the regular DTLZ1-4 problems. As to the irregular DTLZ5-7 problems, RGEA performed better on the DTLZ5 problem with 3 objectives and the DTLZ7 problems with 3 and 5 objectives. As for the irregular WFG1-3 problems, RGEA performed better on the WFG1 problem with 3 objectives, and it also performed better on the WFG2 problems with 3-to-10 objectives. Regarding regular WFG4-9 problems, RGEA performed better on the WFG4-7 problems with 3, 5, and 8 objectives, and it also performed better on the WFG8-9 problems with 3-to-10 objectives.

When RGEA was compared with IBEA, for the regular DTLZ1-4 problems, RGEA performed better on the DTLZ1-2 problems with 3, 5, and 8 objectives, the DTLZ3 problem with 3 objectives, and the DTLZ4 problem with 5 objectives. For irregular DTLZ5-7 problems, RGEA performed better on the DTLZ5-6 problems with 3 objectives. Regarding irregular WFG1-3 problems, RGEA performed better on WFG2 problems with 3 and 5 objectives. RGEA performed better on WFG4-9 problems with 3, 5, and 8 objectives in terms of the regular WFG4-9 problems. Therefore, the effectiveness of the proposed algorithm RGEA on regular and irregular problems was verified.

**Figure 5:**
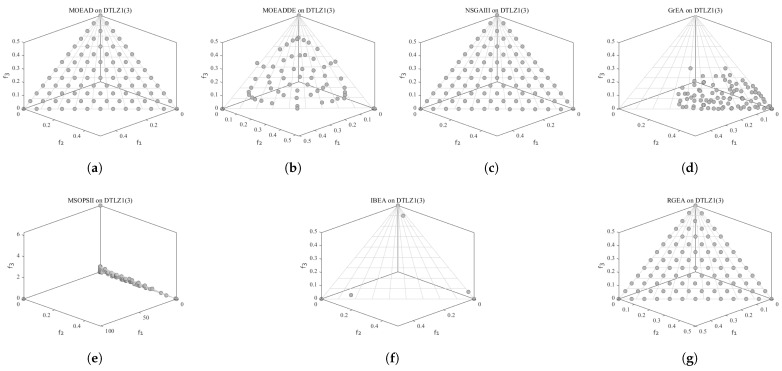
The non-dominated solutions that (**g**) RGEA and (**a**) MOEA/D, (**b**) MOEA/D-DE, (**c**) NSGAIII, (**d**) GrEA, (**e**) MSOPSII, and (**f**) IBEA obtained on the DTLZ1 problem with 3 objectives. The numbers in brackets represent the number of objectives.

**Figure 6:**
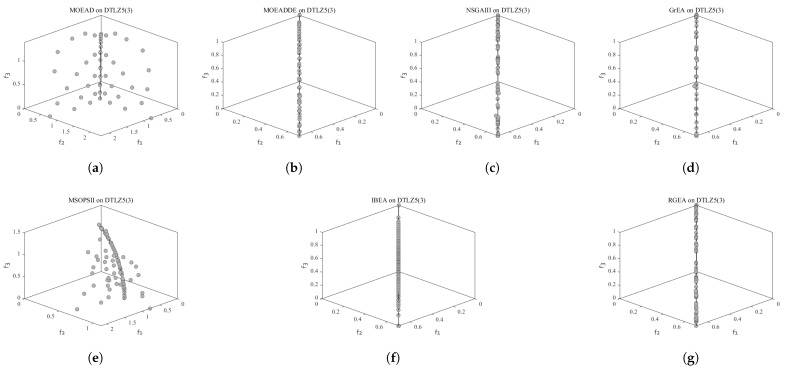
The non-dominated solutions that (**g**) RGEA and (**a**) MOEA/D, (**b**) MOEA/D-DE, (**c**) NSGAIII, (**d**) GrEA, (**e**) MSOPSII, and (**f**) IBEA obtained on the DTLZ5 problem with three objectives. The numbers in brackets represent the number of objectives.

**Figure 7:**
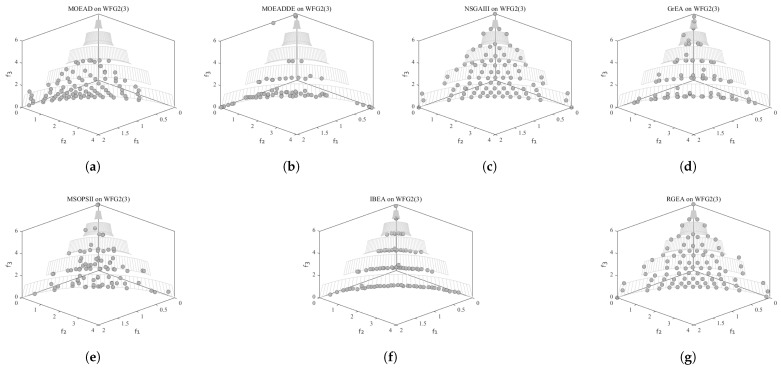
The non-dominated solutions that (**g**) RGEA and (**a**) MOEA/D, (**b**) MOEA/D-DE, (**c**) NSGAIII, (**d**) GrEA, (**e**) MSOPSII, and (**f**) IBEA obtained on the WFG2 problem with three objectives. The numbers in brackets represent the number of objectives.

**Figure 8:**
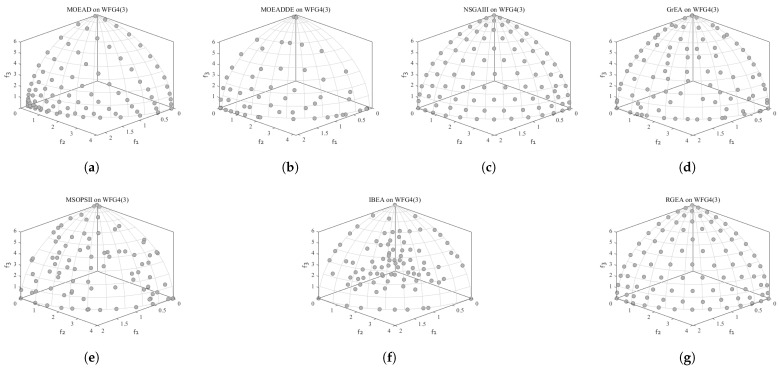
The non-dominated solutions that (**g**) RGEA and (**a**) MOEA/D, (**b**) MOEA/D-DE, (**c**) NSGAIII, (**d**) GrEA, (**e**) MSOPSII, and (**f**) IBEA obtained on the WFG4 problem with three objectives. The numbers in brackets represent the number of objectives.

**Figure 9:**
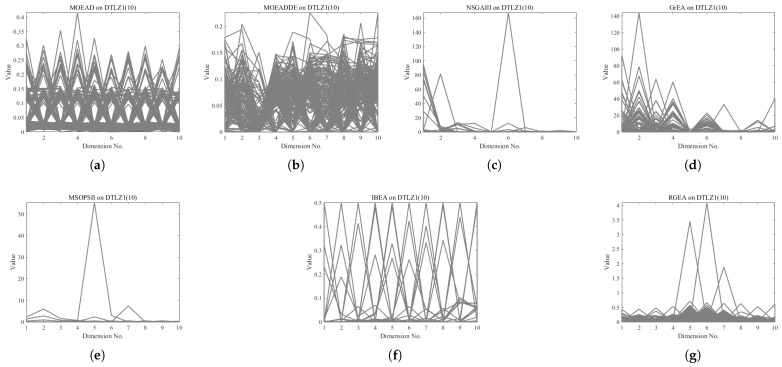
The non-dominated solutions that (**g**) RGEA and (**a**) MOEA/D, (**b**) MOEA/D-DE, (**c**) NSGAIII, (**d**) GrEA, (**e**) MSOPSII, and (**f**) IBEA obtained on the DTLZ1 problem with 10 objectives. The numbers in brackets represent the number of objectives.

**Figure 10:**
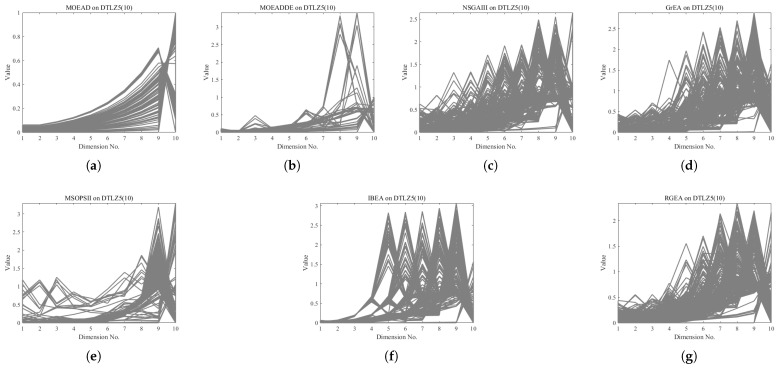
The non-dominated solutions that (**g**) RGEA and (**a**) MOEA/D, (**b**) MOEA/D-DE, (**c**) NSGAIII, (**d**) GrEA, (**e**) MSOPSII, and (**f**) IBEA obtained on the DTLZ5 problem with 10 objectives. The numbers in brackets represent the number of objectives.

**Figure 11:**
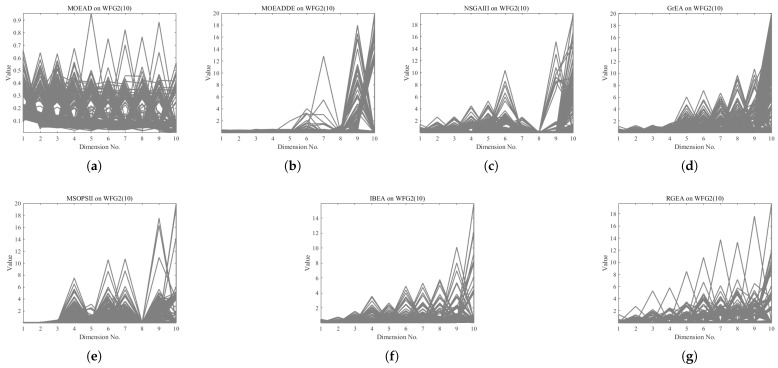
The non-dominated solutions that (**g**) RGEA and (**a**) MOEA/D, (**b**) MOEA/D-DE, (**c**) NSGAIII, (**d**) GrEA, (**e**) MSOPSII, and (**f**) IBEA obtained on the WFG2 problem with 10 objectives. The number in brackets represents the number of objectives.

**Figure 12:**
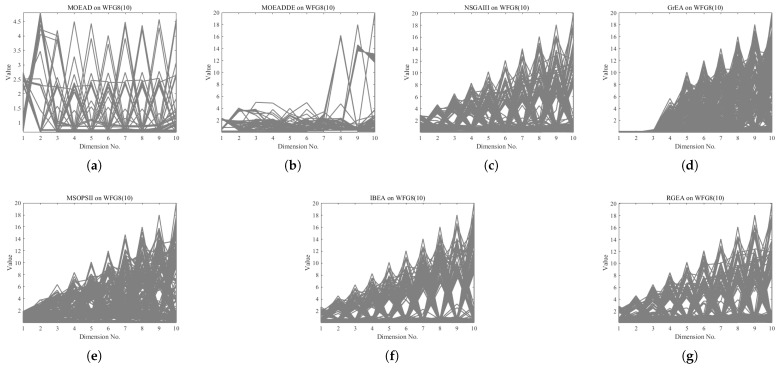
The non-dominated solutions that (**g**) RGEA and (**a**) MOEA/D, (**b**) MOEA/D-DE, (**c**) NSGAIII, (**d**) GrEA, (**e**) MSOPSII, and (**f**) IBEA obtained on the WFG8 problem with 10 objectives. The numbers in brackets represent the number of objectives.

In order to show the non-dominated solutions obtained by RGEA and the other six algorithms without adaptive technology on the regular and irregular problems, we selected the DTLZ1, DTLZ5, WFG2, and WFG4 problems with three objectives as representatives to display (corresponding to Figure 5, Figure 6, Figure 7 and Figure 8), as well as the DTLZ1, DTLZ5, WFG2, and WFG8 problems with 10 objectives as representatives to visualize (corresponding to Figure 9, Figure 10, Figure 11 and Figure 12, respectively). As shown in Figure 5, for the regular three-objective DTLZ1 problem, the non-dominated solution set obtained by RGEA was the best, which is similar to the visualization results of MOEA/D, and it was significantly better than NSGA-III, GrEA, MOEA/D-DE, IBEA, and MSOPSII. As shown in Figure 6, for the irregular DTLZ5 problem with three objectives, RGEA obtained the best non-dominated solution set, which had obvious advantages and was similar to the visualization results of NSGA-III. As shown in Figure 7, for the irregular WFG2 problem with three objectives, RGEA obtained the best non-dominated solution set, which had obvious advantages and was similar to the visualization results of NSGA-III. As shown in Figure 8, RGEA was also able to obtain the best visualization results for the WFG4 problem with 3 regular objectives, which is similar to the visualization results of NSGA-III. This shows that RGEA can obtain better results on the regular and irregular DTLZ and WFG problems with 3 objectives. As shown in Figure 9, for the regular 10-objective DTLZ1 problem, the visualization results of RGEA were better than GrEA and were equivalent to MOEA/D-DE, NSGA-III, and IBEA. The visualization result of MOEA/D was the best because the MOEA/D used a decomposition-based method to decompose the multi-objective problem into multiple single-objective problems, which can find non-dominated solutions well. As shown in Figure 10, for the regular 10-objective DTLZ5 problem, MOEA/D obtained the best distribution of non-dominated solutions, MSOPSII and IBEA had similar effects, and RGEA was better than GrEA (similar to NSGAIII). As shown in Figure 11, for the irregular WFG2 problem with 10 objectives, IBEA obtained the best distribution of non-dominated solutions. RGEA was better than MOEA/D, MOEA/D-DE, and MSOPSII, and it was similar to NSGA-III. As shown in Figure 12, RGEA had obvious advantages over MOEA/D, MOEA/D-DE, GrEA, and MSOPSII for the WFG8 problem with 10 objectives, and it was comparable to IBEA, IDBEA, and NSGA-III. It can be seen that RGEA can obtain better non-dominated solution sets on both regular and irregular problems.

### 5.2. Comparison with Six Evolutionary Algorithms with Adaptive Technology

This section shows the HV values of RGEA and the six evolutionary algorithms with adaptive technology, i.e., as IDBEA, ANSGAIII, MOEA/D-AWA, RVEAa, DEAGNG, and AdaW, on the DTLZ1-7 and WFG1-9 test problems. The experimental results are shown in Table 3. The performance of RGEA was significantly better than IDBEA, ANSGAIII, RVEAa, DEAGNG, and AdaW on all test problems, and it was similar to MOEA/D-AWA. In the HV metric analysis, RGEA had the best performance on the DTLZ1 problem with 3 objectives; the DTLZ4 problem with 5 objectives; the WFG4 problem with 5 objectives; the WFG5 problem with 3, 5, and 8 objectives; the WFG6 problem with 5, 8, and 10 objectives; the WFG7-8 problem with 5 and 8 objectives; and the WFG9 problem with 8 objectives.

When RGEA was compared with IDBEA, RGEA performed better on the DTLZ1 problems with 3, 8, and 10 objectives; on the DTLZ2-3 problems with 3 and 5 objectives; and on the DTLZ4 problem with 5 objectives (with respect to the regular DTLZ1-4 problems). Regarding irregular DTLZ5-7 problems, RGEA performed better on the DTLZ5 problems with 3, 8, and 10 objectives, as well as on the DTLZ7 problem with 3 objectives. Regarding irregular WFG1-3 problems, RGEA performed better on the WFG1 problem with 3 objectives, as well as performed better on the WFG2 problems with 3-to-10 objectives and on the WFG3 problems with 3 and 5 objectives. Concerning regular WFG4-9 problems, RGEA performed better on WFG4-7 problems with 3 and 5 objectives, as well as on the WFG8 problems with 3-to-10 objectives and on the WFG9 problem with 3 objectives.

When RGEA was compared with ANSGAIII, for the regular DTLZ1-4 problems, it performed better on the DTLZ1 and DTLZ3 problems with 3 objectives and on the DTLZ2 problems with 3 and 5 objectives. For the irregular DTLZ5-7 problems, RGEA performed better on the DTLZ5 problem with 8 objectives. For the irregular WFG1-3 problems, RGEA performed better on the WFG1 problem with 5 objectives, as well as on the WFG2 problems with 3 and 5 objectives and on the WFG3 problem with 3 objectives. For the regular WFG4-9 problems, RGEA performed better on the WFG4-7 problems with 3-to-10 objectives, as well as on the WFG8-9 problems with 3, 5, and 8 objectives.

When RGEA was compared with MOEA/D-AWA, RGEA performed better on the DTLZ1 problem with 3 objectives, and it also performs better on the DTLZ2 problem with 8 objectives in terms of regular DTLZ1-4 problems. Concerning irregular DTLZ5-7 problems, RGEA performed better on the DTLZ7 problems with 3 and 5 objectives. Regarding the irregular WFG1-3 problems, RGEA performed better on the WFG2 problem with 3 objectives. Concerning regular WFG4-9 problems, RGEA performed better on the WFG4 problems with 3, 5, and 8 objectives, and it also performed better on the WFG5-9 problems with 3-to-10 objectives.

When RGEA was compared with RVEAa, it performed better on the DTLZ1 and DTLZ3 problems with 3 objectives, the DTLZ2 problems with 3-to-10 objectives and the DTLZ4 problems with 3 and 5 objectives (with respect to the regular DTLZ1-4 problems). For the irregular DTLZ5-7 problems, RGEA performed better on the DTLZ7 problem with 8 objectives. Regarding irregular WFG1-3 problems, RGEA performed better on the WFG1-2 problems with 3-to-10 objectives, and it also performed better on the WFG3 problem with 5 objectives. Concerning regular WFG4-9 problems, RGEA performed well on all problems with 3-to-10 objectives.

When RGEA was compared with DEAGNG, for the regular DTLZ1-4 problems, RGEA performed better on the DTLZ1 problem with 3, 5, and 8 objectives, on the DTLZ2 problem with 3-to-10 objectives, on the DTLZ3 problem with 3 and 5 objectives, and on the DTLZ4 problem with 5 objectives. For the irregular DTLZ5-7 problem, RGEA performed better on the DTLZ5 problem with 8 objectives, and it also performed better on the DTLZ7 problem with 3 objectives. For the irregular WFG1-3 problems, RGEA performed better on the WFG2 problem with 3–10 objectives. For the regular WFG4-9 problem, RGEA performed better on the WFG4 and WFG6-8 problems with 5, 8, and 10 objectives. The performance was better on the WFG5 problem with 3–10 objectives, and the performance was better on the WFG9 problem with 8 and 10 objectives.

When RGEA was compared with AdaW, for the regular DTLZ1-4 problems, RGEA performed better on the DTLZ1 problem with 3, 8, and 10 objectives, and it also performed better on the DTLZ2 and DTLZ4 problems with 5, 8, and 10 objectives. For the irregular DTLZ5-7 problems, RGEA performed better on the DTLZ5 problem with 5, 8, and 10 objectives, and it also performed better on the DTLZ7 problem with 8 and 10 objectives. For irregular WFG1-3 problems, RGEA performed better on WFG2 problems with 10 objectives, and it also performed better on WFG3 problems with 5 objectives. For the regular WFG4-9 problems, RGEA performed better on WFG4-5 and WFG9 problems with 3-to-10 objectives, and it also performed better on WFG6-8 problems with 5, 8, and 10 objectives. Therefore, the effectiveness of the proposed algorithm RGEA on regular and irregular problems was verified.

**Figure 13:**
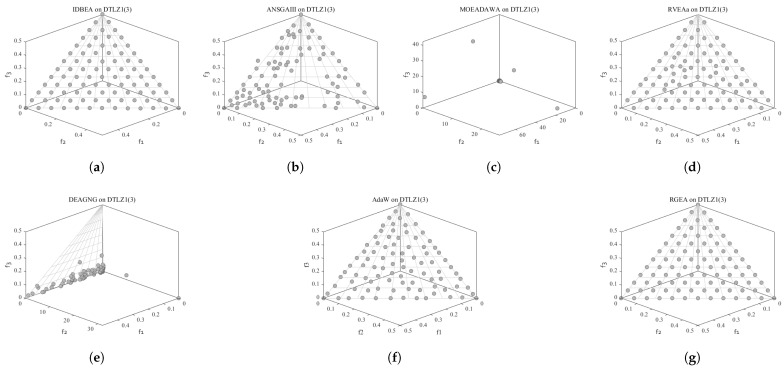
The non-dominated solutions that (**g**) RGEA and (**a**) I-DBEA, (**b**) ANSGAIII, (**c**) MOEA/D-AWA, (**d**) RVEAa, (**e**) DEAGNG, and (**f**) AdaW obtained on the DTLZ1 problem with 3 objectives. The numbers in brackets represent the number of objectives.

**Figure 14:**
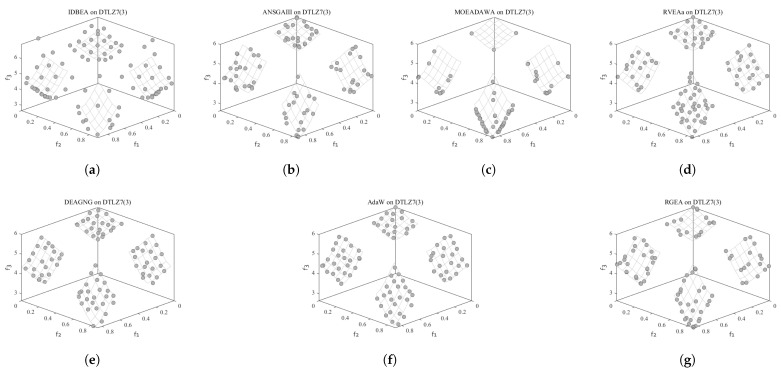
The non-dominated solutions that (**g**) RGEA and (**a**) I-DBEA, (**b**) ANSGAIII, (**c**) MOEA/D-AWA, (**d**) RVEAa, (**e**) DEAGNG, and (**f**) AdaW obtained on the DTLZ7 problem with 3 objectives. The numbers in brackets represent the number of objectives.

**Figure 15:**
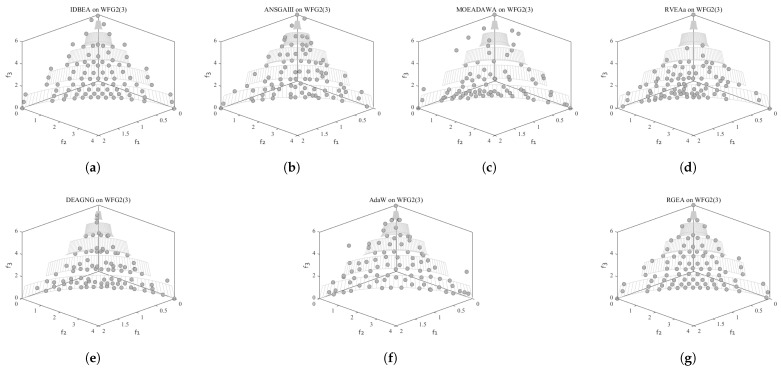
The non-dominated solutions that (**g**) RGEA and (**a**) I-DBEA, (**b**) ANSGAIII, (**c**) MOEA/D-AWA, (**d**) RVEAa, (**e**) DEAGNG, and (**f**) AdaW obtained on the WFG2 problem with 3 objectives. The numbers in brackets represent the number of objectives.

**Figure 16:**
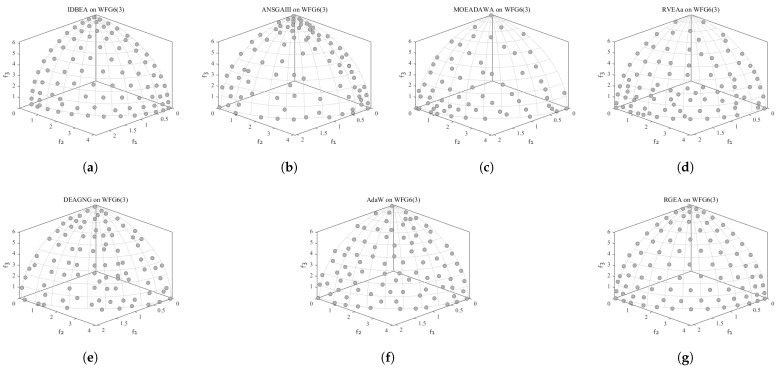
The non-dominated solutions that (**g**) RGEA and (**a**) I-DBEA, (**b**) ANSGAIII, (**c**) MOEA/D-AWA, (**d**) RVEAa, (**e**) DEAGNG, and (**f**) AdaW obtained on the WFG6 problem with 3 objectives. The numbers in brackets represent the number of objectives.

**Figure 17:**
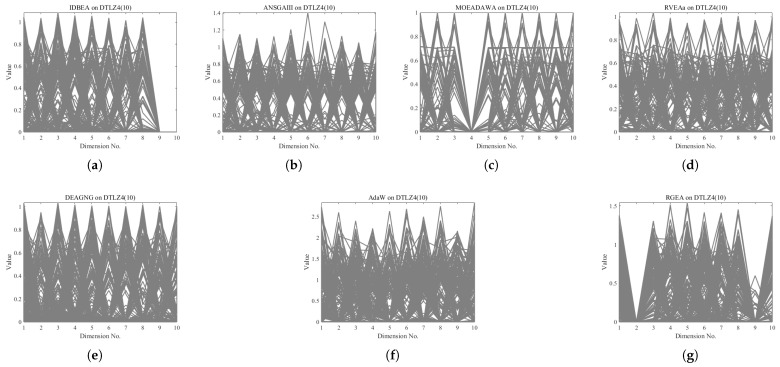
The non-dominated solutions that (**g**) RGEA and (**a**) I-DBEA, (**b**) ANSGAIII, (**c**) MOEA/D-AWA, (**d**) RVEAa, (**e**) DEAGNG, and (**f**) AdaW obtained on the DTLZ4 problem with 10 objectives. The numbers in brackets represent the number of objectives.

**Figure 18:**
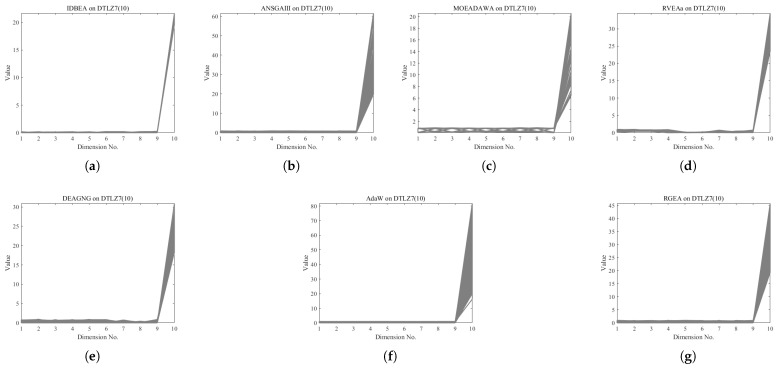
The non-dominated solutions that (**g**) RGEA and (**a**) I-DBEA, (**b**) ANSGAIII, (**c**) MOEA/D-AWA, (**d**) RVEAa, (**e**) DEAGNG, and (**f**) AdaW obtained on the DTLZ7 problem with 10 objectives. The numbers in brackets represent the number of objectives.

**Figure 19:**
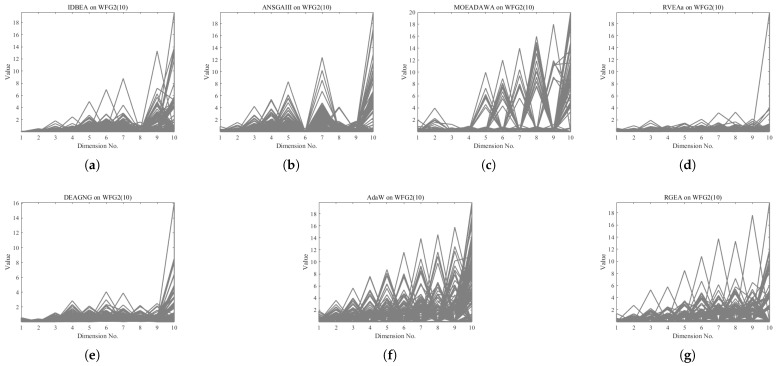
The non-dominated solutions that (**g**) RGEA and (**a**) I-DBEA, (**b**) ANSGAIII, (**c**) MOEA/D-AWA, (**d**) RVEAa, (**e**) DEAGNG, and (**f**) AdaW obtained on the WFG2 problem with 10 objectives. The numbers in brackets represent the number of objectives.

**Figure 20:**
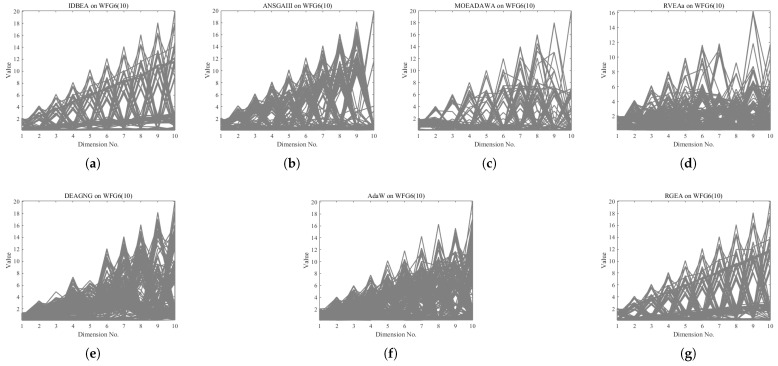
The non-dominated solutions that (**g**) RGEA and (**a**) I-DBEA, (**b**) ANSGAIII, (**c**) MOEA/D-AWA, (**d**) RVEAa, (**e**) DEAGNG, and (**f**) AdaW obtained on the WFG6 problem with 10 objectives. The numbers in brackets represent the number of objectives.

In order to show the non-dominated solution sets obtained by RGEA and the other six algorithms with adaptive technology on regular and irregular problems, we selected DTLZ1, DTLZ7, WFG2, and WFG6 problems with three objectives as representatives for visualization (corresponding to Figure 13, Figure 14, Figure 15 and Figure 16), as well as the DTLZ4, DTLZ7, WFG2, and WFG6 problems with 10 objectives (corresponding to Figure 17, Figure 18, Figure 19 and Figure 20, respectively). As shown in Figure 13, for the regular three-objective DTLZ1 problem, RGEA obtained the best non-dominated solution set and was significantly better than the other six algorithms. As shown in Figure 14, for the irregular DTLZ7 problem with three objectives, AdaW obtained the best visualization results, and the non-dominated solutions obtained by RGEA were significantly better than IDBEA, MOEA/D-AWA, and DEAGNG, which were similar to those of ANSGAIII. As shown in Figure 15, for the irregular WFG2 problem with 3 objectives, the visualization results obtained by RGEA were significantly better than those of IDBEA, ANSGAIII, MOEA/D-AWA, RVEAa, and DEAGNG, but they were similar to those of AdaW. As shown in Figure 16, for the regular three-objective WFG6 problem, the visualization results obtained by RGEA were significantly better than those of IDBEA, ANSGAIII, MOEA/D-AWA, and RVEAa, but they were similar to those of DEAGNG and AdaW. As shown in Figure 17, for the regular 10-objective DTLZ4 problem, the RGEA visualization results were significantly better than AdaW and ANSGAIII. Although the IDBEA visualization results were similar, this did not apply for RVEAa, MOEA/D-AWA and DEAGNG. As shown in Figure 18, for the irregular DTLZ7 problem with 10 objectives, RGEA had obvious advantages over AdaW and was similar to ANSGAIII, but it was not as good as IDBEA, MOEA/D-AWA, RVEAa, and DEAGNG. As shown in Figure 19, for the irregular WFG2 problem with 10 objectives, the visualization results obtained by RGEA were better than IDBEA, RVEAa, DEAGNG, and AdaW, but they were similar to ANSGAIII and slightly inferior to MOEA/D-AWA. As shown in Figure 20, RGEA was similar to IDBEA for the regular WFG6 problem with 10 objectives but had obvious advantages over the other algorithms. It can be seen that RGEA can obtain better non-dominated solution sets on both regular and irregular problems.

### 5.3. Analysis of Parameter Sensitivity

**Figure 21:**
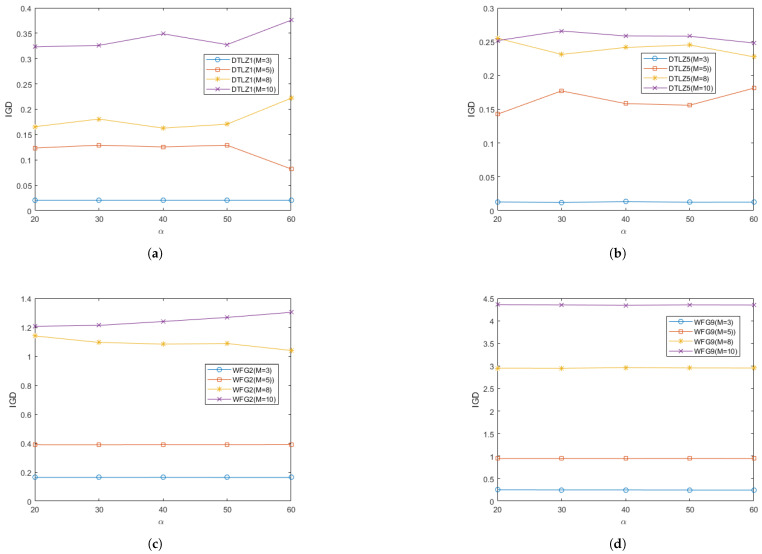
The effect of different values of the parameter α on the IGD values obtained by RGEA for the (**a**) DTLZ1, (**b**) DTLZ5, (**c**) WFG2, and (**d**) WFG9 problems with 3, 5, 8, and 10 objectives. The numbers in brackets represent the number of objectives.

**Figure 22:**
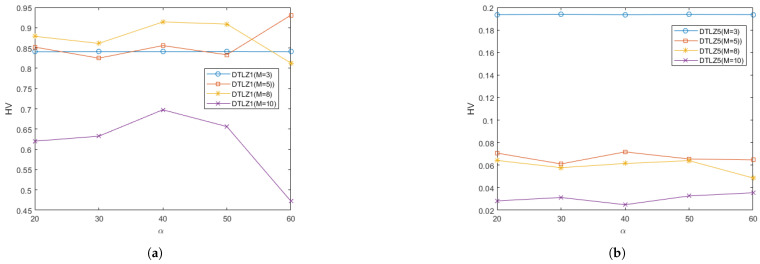

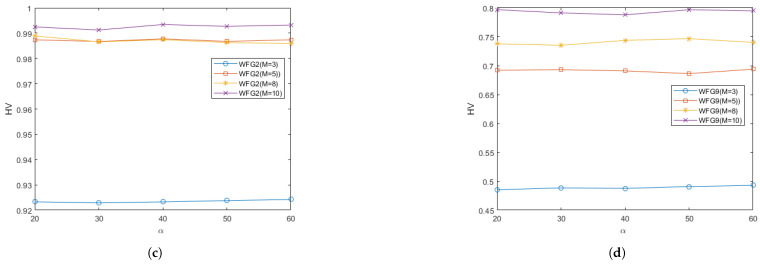
The effect of different values of the parameter α on the HV values obtained by RGEA for the (**a**) DTLZ1, (**b**) DTLZ5, (**c**) WFG2, and (**d**) WFG9 problems with 3, 5, 8, and 10 objectives. The numbers in brackets represent the number of objectives.

In order to study the effect of different values of the parameter α on the performance of RGEA in this paper, two regular problems and two irregular problems were selected for testing: the DTLZ1, DTLZ5, WFG2, and WFG9 test problems. Each test instance ran 20 times independently. The curves of the average IGD and HV metrics obtained by the proposed algorithm RGEA on each test function with different values of the parameter α are shown in Figure 21 and Figure 22. From Figure 21 and Figure 22, it can be seen that the different values of parameter α had little effect on the regular DTLZ1 problem with 3 objectives, the regular WFG9 problems with 3-to-10 objectives, the irregular DTLZ5 problem with 3 objectives, and the irregular WFG2 problems with 3 and 5 objectives. The parameter α had a significant impact on the regular DTLZ1 problems with 5, 8, and 10 objectives, and it had a great impact on the irregular DTLZ5 problems with 5, 8, and 10 objectives. It had a slight impact on the irregular WFG2 problems with 8 and 10 objectives, which indicates that the parameter α did not have much impact on the regular and irregular problems with low-dimensional objectives but that it did have a greater impact on the irregular problems with high-dimensional objectives. According to the analysis of the change curve, the parameter α can achieve relatively good performance at 50, where 50 refers to the entropy value calculated once every 50 iterations. The parameters α control the interval of the calculated entropy and determine the switching time of the reference point-based method and the grid-based method. When the parameters α are different, the distribution of offspring generated by the initial reference point-based environment selection method will also be different, which will directly affect the subsequent grid-based environment selection method, thus affecting the entire population. By introducing entropy and parameter α to determine the switching time of the environment selection method, the current entropy reached the maximum entropy, that is, after determining the number of iterations affected by the parameter α, the distribution of the population would be the best at that time, and the grid-based method was then switched to accelerate local convergence.

## 6. Conclusions

In order to solve the problem where the reference point-based method does not obtain good performance on irregular problems and the grid-based method does not obtain good results on regular problems, this paper proposes the Reference Point and Grid Method-based Evolutionary Algorithm (RGEA) with entropy for many-objective optimization problems. In this paper, the parameter α was introduced to control the interval for calculating the entropy value. By comparing the current entropy with the maximum entropy, the reference point-based method or the grid-based method was determined. When the current entropy reaches the maximum entropy, the grid-based method is adopted. When the current entropy does not reach the maximum entropy, the reference point-based method is adopted. RGEA was compared with six evolutionary algorithms without adaptive technology, i.e., MOEA/D, MOEA/D-DE, NSGAIII, GrEA, MSOPSII, and IBEA, as well as six advanced evolutionary algorithms with adaptive technology, i.e., IDBEA, ANSGAIII, MOEA/D-AWA, RVEAa, DEAGNG, and AdaW. Widely used test problems, such as the DTLZ1-7 and WFG1-9 problems, were selected, and the two types of problems were divided into regular problems and irregular problems when analyzing the specific performance. As verified, RGEA delivered a significant performance in both regular and irregular problems. The main reason why RGEA can effectively deal with regular and irregular problems is that entropy and the parameter α were introduced into RGEA, which can control the interval of calculation entropy. When RGEA reaches the maximum entropy, the reference point-based method is no longer called; rather, it is converted into a grid-based method, which promotes diversity and convergence. RGEA adopts the reference point-based method to improve the population diversity in the early stage of the search, and it then evolves to a later stage, where the entropy calculation is controlled by the parameter α. When the current entropy value reaches the maximum entropy value, the grid-based method is switched to improve the convergence. We also studied the sensitivity of the parameter α, and we concluded that the parameter α did not have much influence on the regular and irregular problems with low-dimensional objectives, but we did find that it had a greater impact on the irregular problems with high-dimensional objectives. However, this paper only studied the influence of the sensitivity of the parameter α on the performance of the RGEA algorithm when the entropy calculation interval was between 20 and 60 generations. In follow-up studies, the diversity and convergence of the population in the evolution process of RGEA could be analyzed to determine the number of specific evolution iterations with superior performance in the evolution process, so as to determine a better interval of entropy calculation. At the same time, such studies could also help determine the switching time used in the reference point-based method and the grid-based method so that the RGEA algorithm can show better performance. Therefore, the study of the parameter α in the evolution process will be the focus of a future research direction. 

## Figures and Tables

**Table 2:** The IGD metric values of RGEA and the six evolutionary algorithms without adaptive technology on the DTLZ1-7 and WFG1-9 problems with 3–10 objectives, where the best result on each test instance is shown in a blue font.

Problem	MOEAD	MOEADDE	NSGAIII	GrEA	MSOPSII	IBEA	RGEA
DTLZ1	$2.0636\times 10^{-2}$($8.02\times 10^{-5}$)=	$3.0615\times 10^{-2}\left(2.24\times 10^{-4}\right)$-	$2.0649\times 10^{-2}\left(1.14\times 10^{-4}\right)$-	$1.0973\times 10^{-1}\left(9.46\times 10^{-2}\right)$-	$2.7907\times 10^{-2}\left(1.61\times 10-3\right)$-	$1.8535\times 10^{-1}\left(3.19\times 10^{-2}\right)$-	$2.0593\times 10^{-2}$(3.38 × 10^−5^)
	$5.3685\times 10^{-2}$(1.08 × 10^−3^)+	$4.1212\times 10^{-1}\left(3.13\times 10^{-1}\right)$-	$5.4787\times 10^{-2}\left(1.93\times 10^{-3}\right)$+	$1.7864\times 10^{-1}\left(9.80\times 10^{-2}\right)$=	$6.6608\times 10^{-2}\left(2.85\times 10^{-3}\right)$=	$1.8316\times 10^{-1}\left(2.21\times 10^{-2}\right)$-	$1.2905\times 10^{-1}\left(9.25\times 10^{-2}\right)$
	$9.0424\times 10^{-2}$(8.93 × 10^−4^)+	$2.3990\times 10^{-1}\left(1.78\times 10^{-1}\right)$=	$1.8388\times 10^{-1}\left(1.35\times 10^{-1}\right)$=	$3.5759\times 10^{-1}\left(2.58\times 10^{-1}\right)$-	$1.8673\times 10^{-1}\left(1.33\times 10^{-2}\right)$=	$2.5453\times 10^{-1}\left(2.68\times 10^{-2}\right)$-	$1.7050\times 10^{-1}\left(6.54\times 10^{-2}\right)$
	$1.0301\times 10^{-1}$(5.60 × 10^−4^)+	$6.1594\times 10^{-1}\left(5.50\times 10^{-1}\right)$=	$2.8048\times 10^{-1}\left(1.43\times 10^{-1}\right)$=	$4.9748\times 10^{-1}\left(2.01\times 10^{-1}\right)$-	$1.8639\times 10^{-1}\left(1.28\times 10^{-2}\right)$+	$2.3114\times 10^{-1}$($1.91\times 10^{-2}$)=	$3.2751\times 10^{-1}\left(2.23\times 10^{-1}\right)$
DTLZ2	$5.4484\times 10^{-2}$(7.77 × 10^−6^)+	$7.6891\times 10^{-2}\left(8.79\times 10^{-4}\right)$-	$5.4510\times 10^{-2}\left(2.21\times 10^{-5}\right)$=	$6.7093\times 10^{-2}\left(1.26\times 10^{-3}\right)$-	$7.0986\times 10^{-2}\left(1.48\times 10^{-3}\right)$-	$8.3221\times 10^{-2}\left(3.35\times 10^{-3}\right)$-	$5.4502\times 10^{-2}\left(1.47\times 10^{-5}\right)$
	$1.6551\times 10^{-1}$(3.72 × 10^−4^)+	$3.6101\times 10^{-1}\left(2.03\times 10^{-2}\right)$-	$1.6824\times 10^{-1}\left(6.77\times 10^{-4}\right)$=	$1.7222\times 10^{-1}\left(1.68\times 10^{-3}\right)$-	$1.8342\times 10^{-1}\left(4.58\times 10^{-3}\right)$-	$1.9220\times 10^{-1}\left(1.55\times 10^{-3}\right)$-	$1.6790\times 10^{-1}\left(5.93\times 10^{-4}\right)$
	$3.1727\times 10^{-1}$(9.89 × 10^−4^)+	$6.2994\times 10^{-1}\left(3.74\times 10^{-2}\right)$-	$3.5387\times 10^{-1}\left(6.22\times 10^{-2}\right)$-	$3.5549\times 10^{-1}\left(3.32\times 10^{-3}\right)$-	$3.8709\times 10^{-1}\left(1.58\times 10^{-2}\right)$-	$3.6357\times 10^{-1}\left(2.92\times 10^{-3}\right)$-	$3.4649\times 10^{-1}\left(1.12\times 10^{-2}\right)$
	$4.0708\times 10^{-1}$($3.55\times 10^{-3}$)+	$6.8249\times 10^{-1}\left(3.61\times 10^{-2}\right)$-	$4.5580\times 10^{-1}\left(2.84\times 10^{-2}\right)$=	$4.0487\times 10^{-1}$(1.34 × 10^−3^)+	$4.4559\times 10^{-1}\left(9.76\times 10^{-3}\right)$=	$4.2760\times 10^{-1}(2.92\times 10^{-3}$)+	$4.6864\times 10^{-1}$($4.68\times 10^{-2}$)
DTLZ3	$5.8553\times 10^{-2}$($3.72\times 10^{-3})$=	$1.1862\times 10^{+0}$($2.99\times 10^{+0})$-	$5.9579\times 10^{-2}$($4.94\times 10^{-3})$=	$1.1892\times 10^{-1}$($9.95\times 10^{-2})$-	$7.6627\times 10^{-2}$($3.40\times 10^{-3})$-	$4.7948\times 10^{-1}\left(4.56\times 10^{-3}\right)$-	$5.7344\times 10^{-2}$(3.41 × 10^−3^)
	$1.3307\times 10^{+0}\left(1.66\times 10^{+0}\right)$=	$1.2503\times 10^{+1}\left(1.41\times 10^{+1}\right)$-	$1.0144\times 10^{+0}\left(1.17\times 10^{+0}\right)$=	$8.3401\times 10^{-1}\left(4.53\times 10^{-1}\right)$-	$2.4307\times 10^{-1}$(1.64 × 10^−2^)=	$5.9960\times 10^{-1}(3.97\times 10^{-3}$)=	$7.1286\times 10^{-1}\left(8.01\times 10^{-1}\right)$
	$1.1880\times 10^{+0}$($2.14\times 10^{+0})$+	$1.7523\times 10^{+0}$($2.58\times 10^{+0})$+	$6.7323\times 10^{+0}$($4.26\times 10^{+0})$-	$7.5568\times 10^{+0}$($3.91\times 10^{+0})$-	$6.2692\times 10^{-1}$(2.63 × 10^−1^)+	$6.7040\times 10^{-1}(1.16\times 10^{-2}$)+	$4.1288\times 10^{+0}$($2.02\times 10^{+0})$
	$7.4320\times 10^{-1}$($3.79\times 10^{-1}$)+	$7.4310\times 10^{+0}$($1.15\times 10^{+1})$+	$1.5920\times 10^{+1}$($9.20\times 10^{+0})$=	$1.5312\times 10^{+1}$($6.37\times 10^{+0})$=	$3.3068\times 10^{+0}$($1.32\times 10^{+0})$+	$7.2387\times 10^{-1}$(6.91 × 10^−3^)+	$1.5899\times 10^{+1}$($1.16\times 10^{+1})$
DTLZ4	$4.2763\times 10^{-1}\left(3.47\times 10^{-1}\right)$=	$1.6440\times 10^{-1}\left(8.17\times 10^{-2}\right)$+	$2.2087\times 10^{-1}\left(2.74\times 10^{-1}\right)$=	$1.5785\times 10^{-1}\left(2.70\times 10^{-1}\right)$+	$2.9598\times 10^{-1}\left(3.38\times 10^{-1}\right)$-	$8.2128\times 10^{-2}$(1.79 × 10^−3^)+	$2.0065\times 10^{-1}\left(2.29\times 10^{-1}\right)$
	$6.0393\times 10^{-1}\left(2.16\times 10^{-1}\right)$-	$4.0978\times 10^{-1}\left(2.47\times 10^{-2}\right)$-	$1.6924\times 10^{-1}$(9.71 × 10^−4^)=	$1.7539\times 10^{-1}\left(2.35\times 10^{-3}\right)$-	$3.1391\times 10^{-1}\left(1.82\times 10^{-1}\right)$-	$2.0213\times 10^{-1}\left(5.07\times 10^{-2}\right)$-	$1.6966\times 10^{-1}\left(1.35\times 10^{-3}\right)$
	$7.0351\times 10^{-1}\left(1.02\times 10^{-1}\right)$-	$7.0957\times 10^{-1}\left(3.09\times 10^{-2}\right)$-	$3.9524\times 10^{-1}\left(8.33\times 10^{-2}\right)$=	$3.5389\times 10^{-1}$(1.73 × 10^−3^)=	$5.7792\times 10^{-1}\left(1.17\times 10^{-1}\right)$-	$3.8105\times 10^{-1}(4.58\times 10^{-2}$)=	$4.1337\times 10^{-1}\left(9.08\times 10^{-2}\right)$
	$7.4196\times 10^{-1}\left(1.33\times 10^{-1}\right)$-	$7.2403\times 10^{-1}\left(2.39\times 10^{-2}\right)$-	$4.8095\times 10^{-1}\left(1.24\times 10^{-2}\right)$=	$4.1082\times 10^{-1}$(1.23 × 10^−3^)+	$5.4949\times 10^{-1}\left(5.33\times 10^{-2}\right)$-	$4.3203\times 10^{-1}(1.20\times 10^{-2}$)+	$4.9043\times 10^{-1}\left(4.15\times 10^{-2}\right)$
DTLZ5	$3.3575\times 10^{-2}\left(1.27\times 10^{-4}\right)$-	$1.4275\times 10^{-2}\left(1.26\times 10^{-4}\right)$-	$1.3020\times 10^{-2}\left(2.00\times 10^{-3}\right)$=	$2.1668\times 10^{-2}\left(1.41\times 10^{-3}\right)$-	$1.7397\times 10^{-2}\left(3.00\times 10^{-3}\right)$-	$1.7160\times 10^{-2}\left(1.63\times 10^{-3}\right)$-	$1.2354\times 10^{-2}$(1.56 × 10^−3^)
	$2.2368\times 10^{-2}$(4.96 × 10^−4^)+	$3.8742\times 10^{-2}\left(1.46\times 10^{-3}\right)$+	$1.6202\times 10^{-1}\left(5.14\times 10^{-2}\right)$=	$1.3354\times 10^{-1}\left(3.55\times 10^{-2}\right)$=	$2.9521\times 10^{-2}\left(5.76\times 10^{-3}\right)$+	$4.6558\times 10^{-2}(1.29\times 10^{-2}$)+	$1.5596\times 10^{-1}\left(4.98\times 10^{-2}\right)$
	$2.4358\times 10^{-2}$(2.85 × 10^−4^)+	$3.9265\times 10^{-2}\left(4.38\times 10^{-3}\right)$+	$2.1377\times 10^{-1}\left(6.35\times 10^{-2}\right)$=	$2.9311\times 10^{-1}\left(7.41\times 10^{-2}\right)$-	$5.5428\times 10^{-2}\left(1.78\times 10^{-2}\right)$+	$9.6771\times 10^{-2}(2.97\times 10^{-2}$)+	$2.4515\times 10^{-1}\left(8.42\times 10^{-2}\right)$
	$1.8808\times 10^{-2}$(5.56 × 10^−4^)+	$5.0328\times 10^{-2}\left(5.79\times 10^{-3}\right)$+	$2.4824\times 10^{-1}\left(4.50\times 10^{-2}\right)$=	$3.2568\times 10^{-1}\left(4.79\times 10^{-2}\right)$-	$1.2915\times 10^{-1}\left(4.68\times 10^{-2}\right)$+	$1.0134\times 10^{-1}(2.56\times 10^{-2}$)+	$2.5804\times 10^{-1}\left(5.10\times 10^{-2}\right)$
DTLZ6	$3.3733\times 10^{-2}\left(1.86\times 10^{-4}\right)$-	$1.4426\times 10^{-2}$(5.21 × 10^−5^)+	$1.9806\times 10^{-2}\left(3.59\times 10^{-3}\right)$=	$2.2262\times 10^{-2}\left(1.56\times 10^{-4}\right)$-	$1.9739\times 10^{-2}\left(6.46\times 10^{-3}\right)$=	$2.5688\times 10^{-2}\left(3.68\times 10^{-3}\right)$-	$1.8897\times 10^{-2}\left(1.56\times 10^{-3}\right)$
	$2.2627\times 10^{-2}$(9.44 × 10^−4^)+	$3.5085\times 10^{-2}\left(2.92\times 10^{-4}\right)$+	$3.9704\times 10^{-1}\left(1.90\times 10^{-1}\right)$=	$2.1789\times 10^{-1}\left(5.69\times 10^{-2}\right)$+	$4.1210\times 10^{-2}\left(1.64\times 10^{-2}\right)$+	$8.3323\times 10^{-2}(1.59\times 10^{-2}$)+	$4.5861\times 10^{-1}\left(2.04\times 10^{-1}\right)$
	$2.6511\times 10^{-1}\left(6.10\times 10^{-1}\right)$+	$2.7271\times 10^{-2}$(2.76 × 10^−3^)+	$2.7428\times 10^{+0}\left(7.36\times 10^{-1}\right)$=	$2.3846\times 10^{+0}\left(5.53\times 10^{-1}\right)$+	$1.5696\times 10^{+0}\left(1.33\times 10^{+0}\right)$+	$2.1295\times 10^{-1}(8.20\times 10^{-2}$)+	$3.2130\times 10^{+0}\left(9.01\times 10^{-1}\right)$
	$1.9442\times 10^{-2}$(1.49 × 10^−3^)+	$2.7334\times 10^{-2}\left(3.27\times 10^{-3}\right)$+	$5.1851\times 10^{+0}\left(6.66\times 10^{-1}\right)$=	$2.6216\times 10^{+0}\left(2.22\times 10^{-1}\right)$+	$2.5161\times 10^{+0}\left(9.76\times 10^{-1}\right)$+	$1.5722\times 10^{-1}(4.74\times 10^{-2}$)+	$5.3456\times 10^{+0}\left(6.81\times 10^{-1}\right)$
DTLZ7	$1.5427\times 10^{-1}\left(2.92\times 10^{-3}\right)$-	$2.7656\times 10^{-1}\left(1.90\times 10^{-1}\right)$-	$7.7647\times 10^{-2}\left(2.92\times 10^{-3}\right)$=	$8.5464\times 10^{-2}$(4.31 × 10^−3^)+	$1.4863\times 10^{-1}\left(2.13\times 10^{-2}\right)$-	$7.8432\times 10^{-2}(3.86\times 10^{-3}$)=	$9.1019\times 10^{-2}\left(6.31\times 10^{-2}\right)$
	$9.0232\times 10^{-1}\left(2.76\times 10^{-1}\right)$-	$1.1202\times 10^{+0}\left(1.40\times 10^{-1}\right)$-	$3.3175\times 10^{-1}\left(2.06\times 10^{-2}\right)$=	$2.3954\times 10^{-1}$(9.49 × 10^−3^)+	$4.5826\times 10^{-1}\left(5.32\times 10^{-2}\right)$-	$2.6808\times 10^{-1}(5.35\times 10^{-3}$)+	$3.3208\times 10^{-1}\left(1.58\times 10^{-2}\right)$
	$1.7949\times 10^{+0}\left(2.60\times 10^{-1}\right)$-	$1.3422\times 10^{+0}\left(1.35\times 10^{-1}\right)$=	$1.4118\times 10^{+0}\left(2.72\times 10^{-1}\right)$=	$8.4908\times 10^{-1}\left(5.29\times 10^{-2}\right)$+	$1.4196\times 10^{+0}\left(1.58\times 10^{-1}\right)$=	$7.9669\times 10^{-1}$(1.68 × 10^−1^)+	$1.3625\times 10^{+0}\left(3.10\times 10^{-1}\right)$
	$2.3112\times 10^{+0}\left(6.18\times 10^{-1}\right)$+	$1.7561\times 10^{+0}\left(1.30\times 10^{-1}\right)$+	$6.0817\times 10^{+0}\left(1.00\times 10^{+0}\right)$=	$3.3754\times 10^{+0}\left(2.40\times 10^{-1}\right)$+	$5.8854\times 10^{+0}\left(1.23\times 10^{+0}\right)$=	$9.8390\times 10^{-1}$(1.71 × 10^−1^)+	$6.3880\times 10^{+0}\left(1.12\times 10^{+0}\right)$
WFG1	$3.3745\times 10^{-1}\left(3.86\times 10^{-2}\right)$-	$1.3730\times 10^{+0}\left(9.07\times 10^{-2}\right)$-	$2.5008\times 10^{-1}\left(2.65\times 10^{-2}\right)$=	$1.9319\times 10^{-1}\left(1.42\times 10^{-2}\right)$+	$2.8095\times 10^{-1}\left(4.99\times 10^{-2}\right)$-	$1.9056\times 10^{-1}$(1.00 × 10^−2^)+	$2.3969\times 10^{-1}\left(3.77\times 10^{-2}\right)$
	$8.0456\times 10^{-1}\left(2.86\times 10^{-2}\right)$-	$1.8824\times 10^{+0}(1.03\times 10^{-1}$-	$5.1913\times 10^{-1}\left(3.94\times 10^{-2}\right)$=	$5.2317\times 10^{-1}\left(2.70\times 10^{-2}\right)$=	$6.0689\times 10^{-1}\left(2.35\times 10^{-1}\right)$=	$4.2618\times 10^{-1}$(8.99 × 10^−3^)+	$5.2071\times 10^{-1}\left(3.62\times 10^{-2}\right)$
	$1.6040\times 10^{+0}\left(3.76\times 10^{-2}\right)$-	$2.3453\times 10^{+0}\left(1.13\times 10^{-1}\right)$-	$1.1666\times 10^{+0}\left(9.40\times 10^{-2}\right)$=	$1.2190\times 10^{+0}\left(5.12\times 10^{-2}\right)$-	$1.0265\times 10^{+0}\left(1.57\times 10^{-1}\right)$+	$9.3510\times 10^{-1}$(2.61 × 10^−2^)+	$1.1656\times 10^{+0}\left(7.95\times 10^{-2}\right)$
	$1.7591\times 10^{+0}\left(3.91\times 10^{-2}\right)$-	$2.4779\times 10^{+0}\left(6.13\times 10^{-2}\right)$-	$1.2563\times 10^{+0}\left(7.38\times 10^{-2}\right)$=	$1.0814\times 10^{+0}\left(2.74\times 10^{-2}\right)$+	$1.1018\times 10^{+0}\left(1.29\times 10^{-1}\right)$+	$9.8397\times 10^{-1}$(2.55 × 10^−2^)+	$1.2229\times 10^{+0}\left(7.79\times 10^{-2}\right)$
WFG2	$2.6401\times 10^{-1}\left(1.89\times 10^{-2}\right)$-	$3.4807\times 10^{-1}\left(1.88\times 10^{-2}\right)$-	$1.6387\times 10^{-1}\left(1.10\times 10^{-3}\right)$=	$2.3701\times 10^{-1}\left(1.40\times 10^{-2}\right)$-	$2.1149\times 10^{-1}\left(1.09\times 10^{-2}\right)$-	$3.0130\times 10^{-1}\left(6.65\times 10^{-3}\right)$-	$1.6336\times 10^{-1}$(9.63 × 10^−4^)
	$7.6001\times 10^{-1}\left(3.52\times 10^{-2}\right)$-	$1.3448\times 10^{+0}\left(4.97\times 10^{-2}\right)$-	$3.8925\times 10^{-1}$(2.08 × 10^−3^)=	$5.1028\times 10^{-1}\left(1.49\times 10^{-2}\right)$-	$4.4105\times 10^{-1}\left(1.20\times 10^{-2}\right)$-	$4.6201\times 10^{-1}\left(5.11\times 10^{-3}\right)$-	$3.8927\times 10^{-1}\left(2.55\times 10^{-3}\right)$
	$1.7747\times 10^{+0}\left(4.98\times 10^{-2}\right)$-	$1.7712\times 10^{+0}\left(6.23\times 10^{-2}\right)$-	$1.0264\times 10^{+0}\left(2.08\times 10^{-1}\right)$=	$1.0043\times 10^{+0}$(3.90 × 10^−2^)=	$1.3996\times 10^{+0}\left(2.43\times 10^{-1}\right)$-	$1.0345\times 10^{+0}(4.10\times 10^{-2}$)=	$1.0872\times 10^{+0}\left(2.32\times 10^{-1}\right)$
	$1.9040\times 10^{+0}\left(3.42\times 10^{-2}\right)$-	$1.8229\times 10^{+0}\left(8.71\times 10^{-2}\right)$-	$1.3488\times 10^{+0}\left(1.08\times 10^{-1}\right)$=	$1.1759\times 10^{+0}\left(4.32\times 10^{-2}\right)$+	$1.7642\times 10^{+0}\left(2.62\times 10^{-1}\right)$-	$1.0950\times 10^{+0}$(3.36 × 10^−2^)+	$1.2672\times 10^{+0}\left(1.53\times 10^{-1}\right)$
WFG3	$2.0616\times 10^{-1}\left(3.44\times 10^{-2}\right)$-	$3.0885\times 10^{-1}\left(3.75\times 10^{-2}\right)$-	$1.2234\times 10^{-1}\left(1.63\times 10^{-2}\right)$=	$7.9631\times 10^{-2}\left(6.17\times 10^{-3}\right)$+	$1.0987\times 10^{-1}\left(1.25\times 10^{-2}\right)$=	$4.2818\times 10^{-2}$(3.15 × 10^−3^)+	$1.1864\times 10^{-1}\left(1.37\times 10^{-2}\right)$
	$9.6285\times 10^{-1}\left(2.34\times 10^{-1}\right)$-	$1.6262\times 10^{+0}\left(7.57\times 10^{-2}\right)$-	$5.1485\times 10^{-1}\left(4.89\times 10^{-2}\right)$=	$3.8192\times 10^{-1}\left(3.40\times 10^{-2}\right)$+	$9.4711\times 10^{-2}$(1.37 × 10^−2^)+	$1.3393\times 10^{-1}(1.43\times 10^{-2}$)+	$5.0362\times 10^{-1}\left(4.64\times 10^{-2}\right)$
	$3.8747\times 10^{+0}\left(1.56\times 10^{-1}\right)$-	$2.5937\times 10^{+0}\left(1.25\times 10^{-1}\right)$-	$1.2372\times 10^{+0}\left(3.18\times 10^{-1}\right)$=	$1.1133\times 10^{+0}\left(1.16\times 10^{-1}\right)$=	$1.7405\times 10^{-1}$(5.23 × 10^−2^)+	$4.2909\times 10^{-1}(3.55\times 10^{-2}$)+	$1.0939\times 10^{+0}\left(1.00\times 10^{-1}\right)$
	$5.4586\times 10^{+0}\left(1.88\times 10^{-1}\right)$-	$3.4960\times 10^{+0}\left(1.59\times 10^{-1}\right)$-	$1.2299\times 10^{+0}\left(3.16\times 10^{-1}\right)$=	$1.9158\times 10^{+0}\left(2.38\times 10^{-1}\right)$-	$1.7727\times 10^{-1}$(5.77 × 10^−2^)+	$4.0131\times 10^{-1}(2.91\times 10^{-2}$)+	$1.2272\times 10^{+0}\left(3.40\times 10^{-1}\right)$
WFG4	$2.8250\times 10^{-1}\left(7.65\times 10^{-3}\right)$-	$4.0629\times 10^{-1}\left(1.21\times 10^{-2}\right)$-	$2.2926\times 10^{-1}\left(1.52\times 10^{-3}\right)$=	$2.5399\times 10^{-1}\left(5.24\times 10^{-3}\right)$-	$2.7323\times 10^{-1}\left(1.14\times 10^{-2}\right)$-	$3.3293\times 10^{-1}\left(1.14\times 10^{-2}\right)$-	$2.2850\times 10^{-1}$(1.58 × 10^−3^)
	$1.7118\times 10^{+0}\left(8.07\times 10^{-2}\right)$-	$2.9654\times 10^{+0}\left(1.50\times 10^{-1}\right)$-	$9.6513\times 10^{-1}\left(2.45\times 10^{-3}\right)$=	$9.6657\times 10^{-1}\left(7.96\times 10^{-3}\right)$=	$1.0885\times 10^{+0}\left(1.06\times 10^{-2}\right)$-	$1.0958\times 10^{+0}\left(8.84\times 10^{-3}\right)$-	$9.6460\times 10^{-1}$(2.31 × 10^−3^)
	$7.1696\times 10^{+0}\left(7.99\times 10^{-2}\right)$-	$5.2902\times 10^{+0}\left(1.77\times 10^{-1}\right)$-	$2.9796\times 10^{+0}\left(8.52\times 10^{-2}\right)$=	$2.8998\times 10^{+0}$(1.75 × 10^−2^)+	$3.2475\times 10^{+0}\left(5.68\times 10^{-2}\right)$-	$3.1981\times 10^{+0}\left(5.14\times 10^{-2}\right)$-	$2.9700\times 10^{+0}\left(3.47\times 10^{-2}\right)$
	$9.4868\times 10^{+0}\left(1.42\times 10^{-1}\right)$-	$7.1531\times 10^{+0}\left(3.33\times 10^{-1}\right)$-	$4.5058\times 10^{+0}\left(7.81\times 10^{-2}\right)$=	$4.1007\times 10^{+0}$(5.04 × 10^−2^)+	$4.3603\times 10^{+0}\left(5.52\times 10^{-2}\right)$+	$4.3716\times 10^{+0}(5.77\times 10^{-2}$)+	$4.5076\times 10^{+0}\left(7.11\times 10^{-2}\right)$
WFG5	$2.6498\times 10^{-1}\left(8.28\times 10^{-3}\right)$-	$3.3674\times 10^{-1}\left(4.99\times 10^{-3}\right)$-	$2.3454\times 10^{-1}$(1.02 × 10^−3^)=	$2.7081\times 10^{-1}\left(9.06\times 10^{-3}\right)$-	$2.9488\times 10^{-1}\left(1.00\times 10^{-2}\right)$-	$3.3574\times 10^{-1}\left(1.63\times 10^{-2}\right)$-	$2.3466\times 10^{-1}\left(1.10\times 10^{-3}\right)$
	$1.6334\times 10^{+0}\left(5.90\times 10^{-2}\right)$-	$2.9975\times 10^{+0}\left(1.48\times 10^{-1}\right)$-	$9.4995\times 10^{-1}$(4.05 × 10^−3^)=	$9.6696\times 10^{-1}\left(9.02\times 10^{-3}\right)$-	$1.0921\times 10^{+0}\left(2.06\times 10^{-2}\right)$-	$1.0805\times 10^{+0}\left(1.34\times 10^{-2}\right)$-	$9.5031\times 10^{-1}\left(3.60\times 10^{-3}\right)$
	$6.8041\times 10^{+0}\left(9.15\times 10^{-2}\right)$-	$5.0038\times 10^{+0}\left(1.15\times 10^{-1}\right)$-	$2.9506\times 10^{+0}\left(1.08\times 10^{-2}\right)$=	$2.8973\times 10^{+0}$(1.82 × 10^−2^)+	$3.2880\times 10^{+0}\left(4.49\times 10^{-2}\right)$-	$3.2459\times 10^{+0}\left(2.65\times 10^{-2}\right)$-	$2.9456\times 10^{+0}\left(1.08\times 10^{-2}\right)$
	$9.1557\times 10^{+0}\left(1.20\times 10^{-1}\right)$-	$6.8231\times 10^{+0}\left(2.06\times 10^{-1}\right)$-	$4.4680\times 10^{+0}\left(1.53\times 10^{-2}\right)$=	$4.0582\times 10^{+0}$(2.79 × 10^−2^)+	$4.4373\times 10^{+0}\left(4.01\times 10^{-2}\right)$+	$4.4614\times 10^{+0}(6.61\times 10^{-2}$)=	$4.4870\times 10^{+0}\left(6.31\times 10^{-2}\right)$
WFG6	$3.0374\times 10^{-1}\left(1.62\times 10^{-2}\right)$-	$3.8045\times 10^{-1}\left(5.76\times 10^{-3}\right)$-	$2.4950\times 10^{-1}\left(6.34\times 10^{-3}\right)$=	$2.7402\times 10^{-1}\left(8.37\times 10^{-3}\right)$-	$3.2100\times 10^{-1}\left(1.17\times 10^{-2}\right)$-	$3.4344\times 10^{-1}\left(1.35\times 10^{-2}\right)$-	$2.4893\times 10^{-1}$(4.50 × 10^−3^)
	$1.8870\times 10^{+0}\left(1.18\times 10^{-1}\right)$-	$2.2625\times 10^{+0}\left(3.05\times 10^{-1}\right)$-	$9.6797\times 10^{-1}\left(2.82\times 10^{-3}\right)$=	$9.8075\times 10^{-1}\left(6.42\times 10^{-3}\right)$-	$1.1555\times 10^{+0}\left(3.24\times 10^{-2}\right)$-	$1.1012\times 10^{+0}\left(1.22\times 10^{-2}\right)$-	$9.6685\times 10^{-1}$(2.92 × 10^−3^)
	$7.4771\times 10^{+0}\left(1.49\times 10^{-1}\right)$-	$5.6090\times 10^{+0}\left(3.78\times 10^{-1}\right)$-	$3.0242\times 10^{+0}\left(1.44\times 10^{-1}\right)$=	$2.9341\times 10^{+0}$(2.16 × 10^−2^)+	$3.3132\times 10^{+0}\left(5.19\times 10^{-2}\right)$-	$3.3187\times 10^{+0}\left(3.35\times 10^{-2}\right)$-	$2.9819\times 10^{+0}\left(1.64\times 10^{-2}\right)$
	$9.7211\times 10^{+0}\left(1.01\times 10^{-1}\right)$-	$7.8005\times 10^{+0}\left(4.12\times 10^{-1}\right)$-	$4.5706\times 10^{+0}\left(1.31\times 10^{-2}\right)$=	$4.0726\times 10^{+0}$(2.50 × 10^−2^)+	$4.4176\times 10^{+0}\left(6.19\times 10^{-2}\right)$+	$4.5800\times 10^{+0}(7.36\times 10^{-2}$)=	$4.5769\times 10^{+0}\left(1.98\times 10^{-2}\right)$
WFG7	$4.3145\times 10^{-1}\left(5.78\times 10^{-2}\right)$-	$4.1496\times 10^{-1}\left(1.95\times 10^{-2}\right)$-	$2.2822\times 10^{-1}$(2.29 × 10^−3^)=	$2.6660\times 10^{-1}\left(1.01\times 10^{-2}\right)$-	$2.8985\times 10^{-1}\left(1.00\times 10^{-2}\right)$-	$3.4067\times 10^{-1}\left(1.09\times 10^{-2}\right)$-	$2.2871\times 10^{-1}\left(1.59\times 10^{-3}\right)$
	$1.8766\times 10^{+0}\left(7.77\times 10^{-2}\right)$-	$3.2667\times 10^{+0}\left(1.59\times 10^{-1}\right)$-	$9.6287\times 10^{-1}\left(4.47\times 10^{-3}\right)$=	$9.8022\times 10^{-1}\left(9.82\times 10^{-3}\right)$-	$1.1470\times 10^{+0}\left(2.22\times 10^{-2}\right)$-	$1.0990\times 10^{+0}\left(1.86\times 10^{-2}\right)$-	$9.6225\times 10^{-1}$(2.43 × 10^−3^)
	$7.3725\times 10^{+0}\left(1.92\times 10^{-1}\right)$-	$5.3737\times 10^{+0}\left(2.12\times 10^{-1}\right)$-	$2.9904\times 10^{+0}\left(1.61\times 10^{-2}\right)$=	$2.8998\times 10^{+0}$(1.79 × 10^−2^)+	$3.3532\times 10^{+0}\left(5.34\times 10^{-2}\right)$-	$3.3423\times 10^{+0}\left(3.54\times 10^{-2}\right)$-	$2.9880\times 10^{+0}\left(1.52\times 10^{-2}\right)$
	$9.6070\times 10^{+0}\left(1.45\times 10^{-1}\right)$-	$7.5340\times 10^{+0}\left(2.59\times 10^{-1}\right)$-	$4.5848\times 10^{+0}\left(9.44\times 10^{-2}\right)$=	$4.0924\times 10^{+0}$(3.71 × 10^−2^)+	$4.4602\times 10^{+0}\left(7.94\times 10^{-2}\right)$+	$4.5363\times 10^{+0}(5.22\times 10^{-2}$)+	$4.5647\times 10^{+0}\left(1.70\times 10^{-2}\right)$
WFG8	$3.1844\times 10^{-1}\left(1.31\times 10^{-2}\right)$-	$5.0018\times 10^{-1}\left(1.98\times 10^{-2}\right)$-	$2.6352\times 10^{-1}\left(4.42\times 10^{-3}\right)$=	$2.8692\times 10^{-1}\left(1.08\times 10^{-2}\right)$-	$3.6843\times 10^{-1}\left(1.14\times 10^{-2}\right)$-	$3.3862\times 10^{-1}\left(9.58\times 10^{-3}\right)$-	$2.6316\times 10^{-1}$(4.22 × 10^−3^)
	$1.5950\times 10^{+0}\left(8.44\times 10^{-2}\right)$-	$3.1911\times 10^{+0}\left(7.94\times 10^{-2}\right)$-	$9.6282\times 10^{-1}$(3.23 × 10^−3^)=	$9.8697\times 10^{-1}\left(9.14\times 10^{-3}\right)$-	$1.1513\times 10^{+0}\left(1.99\times 10^{-2}\right)$-	$1.0830\times 10^{+0}\left(8.65\times 10^{-3}\right)$-	$9.6414\times 10^{-1}\left(2.71\times 10^{-3}\right)$
	$6.4826\times 10^{+0}\left(1.49\times 10^{-1}\right)$-	$5.5297\times 10^{+0}\left(2.30\times 10^{-1}\right)$-	$3.2304\times 10^{+0}\left(2.21\times 10^{-1}\right)$-	$3.0233\times 10^{+0}$(1.91 × 10^−2^)+	$3.4293\times 10^{+0}\left(4.37\times 10^{-2}\right)$-	$3.2277\times 10^{+0}\left(2.83\times 10^{-2}\right)$-	$3.1251\times 10^{+0}\left(1.43\times 10^{-1}\right)$
	$8.7096\times 10^{+0}\left(1.49\times 10^{-1}\right)$-	$7.4329\times 10^{+0}\left(2.39\times 10^{-1}\right)$-	$4.6092\times 10^{+0}\left(2.06\times 10^{-1}\right)$=	$4.9592\times 10^{+0}\left(2.35\times 10^{-1}\right)$-	$4.7273\times 10^{+0}\left(6.76\times 10^{-2}\right)$-	$4.5010\times 10^{+0}$(5.62 × 10^−2^)=	$4.5240\times 10^{+0}\left(2.28\times 10^{-1}\right)$
WFG9	$4.2137\times 10^{-1}\left(5.25\times 10^{-2}\right)$-	$3.5662\times 10^{-1}\left(7.78\times 10^{-3}\right)$-	$2.5519\times 10^{-1}\left(1.33\times 10^{-2}\right)$-	$2.6314\times 10^{-1}\left(1.20\times 10^{-2}\right)$-	$2.7358\times 10^{-1}\left(1.11\times 10^{-2}\right)$-	$3.1593\times 10^{-1}\left(1.22\times 10^{-2}\right)$-	$2.4687\times 10^{-1}$(1.31 × 10^−2^)
	$1.7165\times 10^{+0}\left(8.48\times 10^{-2}\right)$-	$2.8168\times 10^{+0}\left(1.93\times 10^{-1}\right)$-	$9.4481\times 10^{-1}$(7.57 × 10^−3^)=	$9.4806\times 10^{-1}\left(9.52\times 10^{-3}\right)$=	$1.0677\times 10^{+0}\left(4.53\times 10^{-2}\right)$-	$1.0195\times 10^{+0}\left(1.03\times 10^{-2}\right)$-	$9.4825\times 10^{-1}\left(7.52\times 10^{-3}\right)$
	$6.8072\times 10^{+0}\left(1.23\times 10^{-1}\right)$-	$4.9059\times 10^{+0}\left(2.53\times 10^{-1}\right)$-	$2.9637\times 10^{+0}\left(3.92\times 10^{-2}\right)$=	$2.9468\times 10^{+0}$(2.34 × 10^−2^)+	$3.2447\times 10^{+0}\left(4.72\times 10^{-2}\right)$-	$3.0215\times 10^{+0}\left(2.16\times 10^{-2}\right)$-	$2.9555\times 10^{+0}\left(1.47\times 10^{-2}\right)$
	$9.0326\times 10^{+0}\left(2.20\times 10^{-1}\right)$-	$6.7743\times 10^{+0}\left(2.99\times 10^{-1}\right)$-	$4.3437\times 10^{+0}\left(3.58\times 10^{-2}\right)$=	$4.1512\times 10^{+0}\left(3.08\times 10^{-2}\right)$+	$4.4353\times 10^{+0}\left(6.07\times 10^{-2}\right)$-	$4.1359\times 10^{+0}$(2.78 × 10^−2^)+	$4.3535\times 10^{+0}\left(3.92\times 10^{-2}\right)$
+/-/=	16/44/4	11/50/3	1/5/58	26/29/9	18/37/9	26/30/8	

**Table 3:** The HV metric values of RGEA and the six evolutionary algorithms with adaptive technology on the DTLZ1-7 and WFG1-9 problems with 3–10 objectives, where the best result on each test instance is shown in a blue font.

Problem	IDBEA	ANSGAIII	MOEADAWA	RVEAa	DEAGNG	AdaW	RGEA
DTLZ1	$8.3946\times 10^{-1}$$\left(1.75\times 10^{-3}\right)$ -	$8.2799\times 10^{-1}\left(1.11\times 10^{-2}\right)$-	$8.3927\times 10^{-1}\left(2.25\times 10^{-3}\right)$-	$8.3810\times 10^{-1}\left(1.68\times 10^{-3}\right)$-	$7.8097\times 10^{-1}\left(6.57\times 10^{-2}\right)$-	$8.4022\times 10^{-1}\left(4.86\times 10^{-4}\right)$-	$8.4123\times 10^{-1}$(4.31 × 10^−4^)
	$6.8671\times 10^{-1}\left(3.17\times 10^{-1}\right)$=	$9.7553\times 10^{-1}\left(2.94\times 10^{-3}\right)$=	$9.7659\times 10^{-1}\left(4.64\times 10^{-3}\right)$+	$9.7591\times 10^{-1}\left(1.27\times 10^{-3}\right)$=	$7.5795\times 10^{-1}\left(1.49\times 10^{-1}\right)$-	$9.7685\times 10^{-1}$(1.45 × 10^−3^)+	$8.3326\times 10^{-1}\left(2.07\times 10^{-1}\right)$
	$9.8116\times 10^{-2}\left(2.11\times 10^{-1}\right)$-	$8.7078\times 10^{-1}\left(2.01\times 10^{-1}\right)$=	$9.8664\times 10^{-1}\left(6.24\times 10^{-3}\right)$=	$9.8832\times 10^{-1}$(9.50 × 10^−3^)+	$6.5938\times 10^{-1}\left(1.65\times 10^{-1}\right)$-	$5.7572\times 10^{-1}\left(4.02\times 10^{-1}\right)$-	$9.0896\times 10^{-1}\left(1.11\times 10^{-1}\right)$
	$3.7362\times 10^{-1}\left(2.81\times 10^{-1}\right)$-	$6.6221\times 10^{-1}\left(3.59\times 10^{-1}\right)$=	$9.8874\times 10^{-1}$(4.97 × 10^−3^)+	$9.8141\times 10^{-1}\left(4.66\times 10^{-2}\right)$+	$8.4432\times 10^{-1}\left(1.59\times 10^{-1}\right)$=	$9.7554\times 10^{-3}\left(4.36\times 10^{-2}\right)$-	$6.5628\times 10^{-1}(3.84\times 10^{-1}$)
DTLZ2	$5.5809\times 10^{-1}\left(6.88\times 10^{-4}\right)$-	$5.5346\times 10^{-1}\left(3.20\times 10^{-3}\right)$-	$5.6068\times 10^{-1}$(5.68 × 10^−4^)+	$5.5660\times 10^{-1}\left(7.38\times 10^{-4}\right)$-	$5.4560\times 10^{-1}\left(5.21\times 10^{-3}\right)$-	$5.5912\times 10^{-1}\left(7.19\times 10^{-4}\right)$=	$5.5906\times 10^{-1}\left(9.21\times 10^{-5}\right)$
	$7.8623\times 10^{-1}\left(4.86\times 10^{-3}\right)$-	$7.8771\times 10^{-1}\left(2.49\times 10^{-3}\right)$-	$7.9664\times 10^{-1}$(1.21 × 10^−2^)+	$7.8328\times 10^{-1}\left(3.37\times 10^{-3}\right)$-	$7.2741\times 10^{-1}\left(9.67\times 10^{-3}\right)$-	$7.7910\times 10^{-1}\left(4.83\times 10^{-3}\right)$-	$7.9430\times 10^{-1}\left(2.20\times 10^{-3}\right)$
	$9.0225\times 10^{-1}$(2.95 × 10^−3^)+	$8.6895\times 10^{-1}\left(4.29\times 10^{-2}\right)$=	$8.6317\times 10^{-1}\left(1.90\times 10^{-2}\right)$-	$8.7824\times 10^{-1}\left(9.47\times 10^{-3}\right)$-	$7.8847\times 10^{-1}\left(2.06\times 10^{-2}\right)$-	$4.0826\times 10^{-1}\left(9.08\times 10^{-2}\right)$-	$8.8779\times 10^{-1}\left(1.23\times 10^{-2}\right)$
	$9.3911\times 10^{-1}$(4.30 × 10^−3^)+	$8.8247\times 10^{-1}\left(4.10\times 10^{-2}\right)$=	$8.9734\times 10^{-1}\left(2.51\times 10^{-2}\right)$=	$8.7266\times 10^{-1}\left(1.76\times 10^{-2}\right)$-	$8.9248\times 10^{-1}\left(9.58\times 10^{-3}\right)$-	$9.6777\times 10^{-2}\left(1.99\times 10^{-2}\right)$-	$9.0519\times 10^{-1}\left(3.00\times 10^{-2}\right)$
DTLZ3	$3.4712\times 10^{-1}\left(2.11\times 10^{-1}\right)$-	$5.2622\times 10^{-1}\left(1.75\times 10^{-2}\right)$-	$5.4924\times 10^{-1}$(6.83 × 10^−3^)=	$5.3268\times 10^{-1}\left(1.05\times 10^{-2}\right)$-	$4.8172\times 10^{-1}\left(3.49\times 10^{-2}\right)$-	$5.4564\times 10^{-1}\left(1.09\times 10^{-2}\right)$=	$5.4327\times 10^{-1}\left(1.09\times 10^{-2}\right)$
	$2.1447\times 10^{-2}\left(5.24\times 10^{-2}\right)$-	$4.1205\times 10^{-1}\left(2.82\times 10^{-1}\right)$=	$7.6092\times 10^{-1}$(4.28 × 10^−2^)+	$5.8534\times 10^{-1}\left(2.76\times 10^{-1}\right)$+	$2.3865\times 10^{-1}\left(2.65\times 10^{-1}\right)$-	$2.5494\times 10^{-1}\left(2.25\times 10^{-1}\right)$=	$4.0926\times 10^{-1}\left(3.25\times 10^{-1}\right)$
	$5.4490\times 10^{-2}\left(5.90\times 10^{-2}\right)$+	$5.1518\times 10^{-3}\left(2.30\times 10^{-2}\right)$=	$7.6989\times 10^{-1}$(7.00 × 10^−2^)+	$1.0264\times 10^{-1}\left(2.11\times 10^{-1}\right)$+	$4.2131\times 10^{-2}\left(1.28\times 10^{-1}\right)$=	$0.0000\times 10^{+0}\left(0.00\times 10^{+0}\right)$=	$0.0000\times 10^{+0}\left(0.00\times 10^{+0}\right)$
	$5.7707\times 10^{-3}\left(1.84\times 10^{-2}\right)$=	$0.0000\times 10^{+0}\left(0.00\times 10^{+0}\right)$=	$7.9356\times 10^{-1}$(5.44 × 10^−2^)+	$0.0000\times 10^{+0}\left(0.00\times 10^{+0}\right)$=	$1.4120\times 10^{-4}\left(6.31\times 10^{-4}\right)$=	$0.0000\times 10^{+0}\left(0.00\times 10^{+0}\right)$=	$0.0000\times 10^{+0}\left(0.00\times 10^{+0}\right)$
DTLZ4	$4.9949\times 10^{-1}\left(1.04\times 10^{-1}\right)$+	$5.1327\times 10^{-1}\left(8.96\times 10^{-2}\right)$+	$5.2806\times 10^{-1}$(7.93 × 10^−2^)+	$4.9111\times 10^{-1}\left(1.27\times 10^{-1}\right)$-	$5.4470\times 10^{-1}\left(2.60\times 10^{-2}\right)$+	$5.2707\times 10^{-1}\left(7.76\times 10^{-2}\right)$=	$4.9316\times 10^{-1}\left(1.03\times 10^{-1}\right)$
	$6.7734\times 10^{-1}\left(1.45\times 10^{-1}\right)$-	$7.9248\times 10^{-1}\left(3.50\times 10^{-3}\right)$=	$7.2873\times 10^{-1}\left(9.06\times 10^{-2}\right)$=	$7.7859\times 10^{-1}\left(2.81\times 10^{-2}\right)$-	$7.7668\times 10^{-1}\left(4.34\times 10^{-3}\right)$-	$7.8175\times 10^{-1}\left(6.39\times 10^{-3}\right)$-	$7.9292\times 10^{-1}$(4.48 × 10^−3^)
	$8.4750\times 10^{-1}\left(2.88\times 10^{-2}\right)$=	$8.9007\times 10^{-1}\left(3.90\times 10^{-2}\right)$+	$8.6185\times 10^{-1}\left(6.44\times 10^{-2}\right)$=	$8.9978\times 10^{-1}$(1.21 × 10^−2^)+	$8.9291\times 10^{-1}\left(5.16\times 10^{-3}\right)$=	$2.6883\times 10^{-1}\left(6.57\times 10^{-2}\right)$-	$8.5848\times 10^{-1}\left(6.19\times 10^{-2}\right)$
	$9.1282\times 10^{-1}\left(2.90\times 10^{-2}\right)$=	$9.2440\times 10^{-1}\left(8.79\times 10^{-3}\right)$=	$9.5168\times 10^{-1}\left(1.14\times 10^{-2}\right)$+	$9.4938\times 10^{-1}\left(3.43\times 10^{-3}\right)$+	$9.5936\times 10^{-1}$(1.45 × 10^−3^)+	$9.8556\times 10^{-2}\left(5.90\times 10^{-2}\right)$-	$9.0926\times 10^{-1}\left(6.10\times 10^{-2}\right)$
DTLZ5	$1.9177\times 10^{-1}\left(1.92\times 10^{-3}\right)$-	$1.9522\times 10^{-1}\left(6.58\times 10^{-4}\right)$+	$1.9482\times 10^{-1}\left(4.67\times 10^{-4}\right)$+	$1.9814\times 10^{-1}\left(3.06\times 10^{-4}\right)$+	$1.9980\times 10^{-1}$(1.25 × 10^−4^)+	$1.9962\times 10^{-1}\left(7.53\times 10^{-4}\right)$+	$1.9392\times 10^{-1}\left(7.95\times 10^{-4}\right)$
	$1.1691\times 10^{-1}$(2.66 × 10^−3^)+	$6.1472\times 10^{-2}\left(3.00\times 10^{-2}\right)$=	$1.0262\times 10^{-1}\left(3.79\times 10^{-3}\right)$+	$8.8270\times 10^{-2}\left(1.10\times 10^{-2}\right)$+	$5.7872\times 10^{-2}\left(2.54\times 10^{-2}\right)$=	$3.9238\times 10^{-2}\left(2.05\times 10^{-2}\right)$-	$6.5490\times 10^{-2}\left(2.54\times 10^{-2}\right)$
	$2.1036\times 10^{-2}\left(2.72\times 10^{-2}\right)$-	$4.7509\times 10^{-2}\left(1.83\times 10^{-2}\right)$-	$9.9651\times 10^{-2}$(3.91 × 10^−4^)+	$7.8937\times 10^{-2}\left(7.84\times 10^{-3}\right)$+	$4.0907\times 10^{-2}\left(2.67\times 10^{-2}\right)$-	$8.5315\times 10^{-3}\left(1.74\times 10^{-2}\right)$-	$6.3997\times 10^{-2}\left(1.22\times 10^{-2}\right)$
	$0.0000\times 10^{+0}\left(0.00\times 10^{+0}\right)$-	$2.2407\times 10^{-2}\left(1.74\times 10^{-2}\right)$=	$9.6502\times 10^{-2}$(2.64 × 10^−4^)+	$6.5133\times 10^{-2}\left(9.75\times 10^{-3}\right)$+	$3.1109\times 10^{-2}\left(2.54\times 10^{-2}\right)$=	$6.0735\times 10^{-5}\left(1.95\times 10^{-4}\right)$-	$3.2674\times 10^{-2}\left(2.31\times 10^{-2}\right)$
DTLZ6	$1.9001\times 10^{-1}\left(6.70\times 10^{-3}\right)$=	$1.9375\times 10^{-1}\left(1.26\times 10^{-3}\right)$+	$1.9483\times 10^{-1}\left(4.98\times 10^{-4}\right)$+	$1.9792\times 10^{-1}\left(4.91\times 10^{-4}\right)$+	$1.9980\times 10^{-1}$(1.52 × 10^−4^)+	$1.9978\times 10^{-1}\left(2.69\times 10^{-4}\right)$+	$1.9049\times 10^{-1}\left(1.29\times 10^{-3}\right)$
	$1.6247\times 10^{-2}\left(3.29\times 10^{-2}\right)$=	$2.1315\times 10^{-3}\left(3.84\times 10^{-3}\right)$=	$1.0131\times 10^{-1}$(2.82 × 10^−3^)+	$5.6846\times 10^{-2}\left(4.42\times 10^{-2}\right)$+	$2.1871\times 10^{-2}\left(2.77\times 10^{-2}\right)$+	$1.2126\times 10^{-2}\left(1.84\times 10^{-2}\right)$+	$2.4586\times 10^{-3}\left(4.27\times 10^{-3}\right)$
	$1.7390\times 10^{-2}\left(3.56\times 10^{-2}\right)$+	$0.0000\times 10^{+0}\left(0.00\times 10^{+0}\right)$=	$9.9602\times 10^{-2}$(3.16 × 10^−4^)+	$4.8291\times 10^{-2}\left(4.34\times 10^{-2}\right)$+	$1.9513\times 10^{-3}\left(7.05\times 10^{-3}\right)$+	$0.0000\times 10^{+0}\left(0.00\times 10^{+0}\right)$=	$0.0000\times 10^{+0}\left(0.00\times 10^{+0}\right)$
	$0.0000\times 10^{+0}\left(0.00\times 10^{+0}\right)$=	$0.0000\times 10^{+0}\left(0.00\times 10^{+0}\right)$=	$9.6479\times 10^{-2}$(3.15 × 10^−4^)+	$8.7883\times 10^{-3}\left(2.66\times 10^{-2}\right)$+	$4.5340\times 10^{-3}\left(2.03\times 10^{-2}\right)$+	$0.0000\times 10^{+0}\left(0.00\times 10^{+0}\right)$=	$0.0000\times 10^{+0}\left(0.00\times 10^{+0}\right)$
DTLZ7	$2.4153\times 10^{-1}\left(5.74\times 10^{-2}\right)$-	$2.6508\times 10^{-1}\left(7.37\times 10^{-3}\right)$=	$2.4796\times 10^{-1}\left(9.69\times 10^{-3}\right)$-	$2.7182\times 10^{-1}\left(7.63\times 10^{-3}\right)$+	$2.6218\times 10^{-1}\left(2.23\times 10^{-2}\right)$-	$2.7597\times 10^{-1}$(9.59 × 10^−4^)+	$2.6557\times 10^{-1}\left(7.61\times 10^{-3}\right)$
	$2.2759\times 10^{-1}\left(1.85\times 10^{-2}\right)$+	$2.0953\times 10^{-1}\left(9.73\times 10^{-3}\right)$=	$1.5692\times 10^{-1}\left(1.41\times 10^{-2}\right)$-	$2.0738\times 10^{-1}\left(1.07\times 10^{-2}\right)$=	$2.4812\times 10^{-1}$(4.63 × 10^−3^)+	$2.1097\times 10^{-1}\left(8.16\times 10^{-3}\right)$=	$2.0946\times 10^{-1}\left(7.70\times 10^{-3}\right)$
	$1.7756\times 10^{-1}$(1.42 × 10^−2^)+	$9.4999\times 10^{-2}\left(2.66\times 10^{-2}\right)$=	$1.0273\times 10^{-1}\left(2.12\times 10^{-2}\right)$=	$2.5918\times 10^{-2}\left(2.84\times 10^{-2}\right)$-	$1.6522\times 10^{-1}\left(9.02\times 10^{-3}\right)$+	$1.3554\times 10^{-2}\left(1.51\times 10^{-2}\right)$-	$9.0284\times 10^{-2}\left(2.28\times 10^{-2}\right)$
	$1.3912\times 10^{-1}$(2.52 × 10^−2^)+	$2.5806\times 10^{-3}\left(2.79\times 10^{-3}\right)$=	$4.8417\times 10^{-2}\left(2.80\times 10^{-2}\right)$+	$9.9652\times 10^{-3}\left(1.09\times 10^{-2}\right)$+	$7.2138\times 10^{-2}\left(2.01\times 10^{-2}\right)$+	$1.3528\times 10^{-6}\left(3.20\times 10^{-6}\right)$-	$2.1171\times 10^{-3}\left(3.73\times 10^{-3}\right)$
WFG1	$7.2941\times 10^{-1}\left(3.06\times 10^{-2}\right)$-	$8.5732\times 10^{-1}\left(1.85\times 10^{-2}\right)$=	$9.2556\times 10^{-1}$(6.05 × 10^−3^)+	$8.0062\times 10^{-1}\left(4.21\times 10^{-2}\right)$-	$8.8061\times 10^{-1}\left(2.15\times 10^{-2}\right)$+	$8.8949\times 10^{-1}\left(1.28\times 10^{-2}\right)$+	$8.6309\times 10^{-1}\left(2.47\times 10^{-2}\right)$
	$8.5464\times 10^{-1}\left(3.38\times 10^{-2}\right)$=	$8.1909\times 10^{-1}\left(2.38\times 10^{-2}\right)$-	$9.9567\times 10^{-1}$(8.67 × 10^−3^)+	$7.5256\times 10^{-1}\left(5.61\times 10^{-2}\right)$-	$8.8859\times 10^{-1}\left(2.02\times 10^{-2}\right)$+	$9.3234\times 10^{-1}\left(1.60\times 10^{-2}\right)$+	$8.5018\times 10^{-1}\left(2.17\times 10^{-2}\right)$
	$9.4667\times 10^{-1}\left(5.77\times 10^{-2}\right)$+	$8.1069\times 10^{-1}\left(3.58\times 10^{-2}\right)$+	$9.9901\times 10^{-1}$(4.69 × 10^−4^)+	$7.0406\times 10^{-1}\left(6.91\times 10^{-2}\right)$-	$8.7146\times 10^{-1}\left(4.26\times 10^{-2}\right)$+	$8.3247\times 10^{-1}\left(2.85\times 10^{-2}\right)$+	$7.8199\times 10^{-1}\left(4.30\times 10^{-2}\right)$
	$9.8377\times 10^{-1}\left(2.32\times 10^{-2}\right)$+	$9.2087\times 10^{-1}\left(5.18\times 10^{-2}\right)$=	$9.9997\times 10^{-1}$(1.09 × 10^−5^)+	$8.5371\times 10^{-1}\left(8.05\times 10^{-2}\right)$-	$9.5593\times 10^{-1}\left(4.50\times 10^{-2}\right)$+	$9.1397\times 10^{-1}\left(4.67\times 10^{-2}\right)$=	$9.0388\times 10^{-1}\left(4.22\times 10^{-2}\right)$
WFG2	$9.0564\times 10^{-1}\left(4.67\times 10^{-3}\right)$-	$9.1746\times 10^{-1}\left(3.34\times 10^{-3}\right)$-	$9.1330\times 10^{-1}\left(3.98\times 10^{-3}\right)$-	$9.0976\times 10^{-1}\left(5.79\times 10^{-3}\right)$-	$9.1707\times 10^{-1}\left(5.05\times 10^{-3}\right)$-	$9.2828\times 10^{-1}$(1.79 × 10^−3^)+	$9.2377\times 10^{-1}\left(1.85\times 10^{-3}\right)$
	$9.8434\times 10^{-1}\left(2.06\times 10^{-3}\right)$-	$9.8034\times 10^{-1}\left(3.19\times 10^{-3}\right)$-	$9.8729\times 10^{-1}\left(5.77\times 10^{-3}\right)$=	$9.6360\times 10^{-1}\left(5.47\times 10^{-3}\right)$-	$9.6747\times 10^{-1}\left(5.91\times 10^{-3}\right)$-	$9.9021\times 10^{-1}$(1.63 × 10^−3^)+	$9.8676\times 10^{-1}(1.57\times 10^{-3}$)
	$9.6268\times 10^{-1}\left(8.03\times 10^{-2}\right)$-	$9.8961\times 10^{-1}\left(3.73\times 10^{-3}\right)$=	$9.9162\times 10^{-1}$(2.66 × 10^−3^)+	$9.4128\times 10^{-1}\left(1.19\times 10^{-2}\right)$-	$9.6670\times 10^{-1}\left(8.42\times 10^{-3}\right)$-	$9.8664\times 10^{-1}\left(5.24\times 10^{-3}\right)$=	$9.8627\times 10^{-1}\left(5.79\times 10^{-3}\right)$
	$6.5801\times 10^{-1}\left(3.97\times 10^{-1}\right)$-	$9.9313\times 10^{-1}\left(2.57\times 10^{-3}\right)$=	$9.9665\times 10^{-1}$(8.32 × 10^−4^)+	$9.5035\times 10^{-1}\left(9.57\times 10^{-3}\right)$-	$9.7865\times 10^{-1}\left(3.85\times 10^{-3}\right)$-	$9.8989\times 10^{-1}\left(2.75\times 10^{-3}\right)$-	$9.9269\times 10^{-1}\left(3.34\times 10^{-3}\right)$
WFG3	$3.4019\times 10^{-1}\left(1.32\times 10^{-2}\right)$-	$3.6409\times 10^{-1}\left(1.00\times 10^{-2}\right)$-	$3.9352\times 10^{-1}$(5.54 × 10^−3^)+	$3.7684\times 10^{-1}\left(7.26\times 10^{-3}\right)$=	$3.8494\times 10^{-1}\left(9.26\times 10^{-3}\right)$+	$3.7390\times 10^{-1}\left(6.15\times 10^{-3}\right)$=	$3.7576\times 10^{-1}\left(5.55\times 10^{-3}\right)$
	$6.4880\times 10^{-2}\left(1.96\times 10^{-2}\right)$-	$1.2077\times 10^{-1}\left(1.47\times 10^{-2}\right)$+	$1.7812\times 10^{-1}$(2.28 × 10^−2^)+	$8.3317\times 10^{-2}\left(2.11\times 10^{-2}\right)$-	$9.5118\times 10^{-2}\left(3.24\times 10^{-2}\right)$=	$4.2380\times 10^{-2}\left(1.43\times 10^{-2}\right)$-	$1.0351\times 10^{-1}\left(1.74\times 10^{-2}\right)$
	$7.7280\times 10^{-2}$(5.23 × 10^−3^)+	$5.1654\times 10^{-3}\left(8.00\times 10^{-3}\right)$+	$1.0230\times 10^{-2}\left(2.05\times 10^{-2}\right)$+	$0.0000\times 10^{+0}\left(0.00\times 10^{+0}\right)$=	$1.2319\times 10^{-3}\left(2.58\times 10^{-3}\right)$+	$0.0000\times 10^{+0}\left(0.00\times 10^{+0}\right)$=	$0.0000\times 10^{+0}\left(0.00\times 10^{+0}\right)$
	$6.9999\times 10^{-2}$(7.80 × 10^−3^)+	$0.0000\times 10^{+0}\left(0.00\times 10^{+0}\right)$=	$5.2013\times 10^{-3}\left(1.13\times 10^{-2}\right)$+	$0.0000\times 10^{+0}\left(0.00\times 10^{+0}\right)$=	$0.0000\times 10^{+0}\left(0.00\times 10^{+0}\right)$=	$0.0000\times 10^{+0}\left(0.00\times 10^{+0}\right)$=	$0.0000\times 10^{+0}\left(0.00\times 10^{+0}\right)$
WFG4	$4.9466\times 10^{-1}\left(5.70\times 10^{-3}\right)$-	$5.0185\times 10^{-1}\left(3.07\times 10^{-3}\right)$-	$5.2232\times 10^{-1}\left(6.30\times 10^{-3}\right)$-	$5.1057\times 10^{-1}\left(3.62\times 10^{-3}\right)$-	$5.3044\times 10^{-1}$(3.42 × 10^−3^)=	$5.2766\times 10^{-1}\left(2.49\times 10^{-3}\right)$-	$5.2965\times 10^{-1}\left(2.47\times 10^{-3}\right)$
	$7.3777\times 10^{-1}\left(4.59\times 10^{-3}\right)$-	$7.2954\times 10^{-1}\left(5.77\times 10^{-3}\right)$-	$6.8771\times 10^{-1}\left(1.68\times 10^{-2}\right)$-	$7.0142\times 10^{-1}\left(6.99\times 10^{-3}\right)$-	$7.0446\times 10^{-1}\left(5.71\times 10^{-3}\right)$-	$7.2731\times 10^{-1}\left(4.77\times 10^{-3}\right)$-	$7.5003\times 10^{-1}$(3.58 × 10^−3^)
	$8.3273\times 10^{-1}$(8.48 × 10^−3^)=	$7.9255\times 10^{-1}\left(1.56\times 10^{-2}\right)$-	$7.9898\times 10^{-1}\left(1.31\times 10^{-2}\right)$-	$7.0521\times 10^{-1}\left(2.66\times 10^{-2}\right)$-	$7.3843\times 10^{-1}\left(1.21\times 10^{-2}\right)$-	$7.4466\times 10^{-1}\left(1.23\times 10^{-2}\right)$-	$8.3022\times 10^{-1}\left(7.26\times 10^{-3}\right)$
	$9.0359\times 10^{-1}$(1.32 × 10^−2^)+	$8.5330\times 10^{-1}\left(1.97\times 10^{-2}\right)$-	$8.9039\times 10^{-1}\left(7.67\times 10^{-3}\right)$+	$7.2986\times 10^{-1}\left(1.49\times 10^{-2}\right)$-	$7.9917\times 10^{-1}\left(1.11\times 10^{-2}\right)$-	$7.4724\times 10^{-1}\left(1.46\times 10^{-2}\right)$-	$8.7427\times 10^{-1}\left(9.82\times 10^{-3}\right)$
WFG5	$4.8569\times 10^{-1}\left(5.61\times 10^{-3}\right)$-	$4.8962\times 10^{-1}\left(3.87\times 10^{-3}\right)$-	$4.8387\times 10^{-1}\left(3.81\times 10^{-3}\right)$-	$4.9359\times 10^{-1}\left(4.56\times 10^{-3}\right)$-	$4.9514\times 10^{-1}\left(3.06\times 10^{-3}\right)$-	$5.0336\times 10^{-1}\left(2.72\times 10^{-3}\right)$-	$5.1044\times 10^{-1}$(2.44 × 10^−3^)
	$7.2937\times 10^{-1}\left(3.66\times 10^{-3}\right)$-	$7.1142\times 10^{-1}\left(4.78\times 10^{-3}\right)$-	$7.1723\times 10^{-1}\left(1.24\times 10^{-2}\right)$-	$6.7271\times 10^{-1}\left(5.65\times 10^{-3}\right)$-	$6.6189\times 10^{-1}\left(8.42\times 10^{-3}\right)$-	$7.0741\times 10^{-1}\left(3.41\times 10^{-3}\right)$-	$7.3597\times 10^{-1}$(2.28 × 10^−3^)
	$8.1244\times 10^{-1}\left(9.18\times 10^{-3}\right)$=	$7.6143\times 10^{-1}\left(1.09\times 10^{-2}\right)$-	$7.3306\times 10^{-1}\left(9.86\times 10^{-3}\right)$-	$6.9161\times 10^{-1}\left(1.35\times 10^{-2}\right)$-	$6.5498\times 10^{-1}\left(1.96\times 10^{-2}\right)$-	$7.0152\times 10^{-1}\left(1.38\times 10^{-2}\right)$-	$8.1657\times 10^{-1}$(3.85 × 10^−3^)
	$8.6013\times 10^{-1}$(4.11 × 10^−3^)=	$8.0829\times 10^{-1}\left(8.15\times 10^{-3}\right)$-	$8.2101\times 10^{-1}\left(6.08\times 10^{-3}\right)$-	$7.2514\times 10^{-1}\left(1.30\times 10^{-2}\right)$-	$7.4531\times 10^{-1}\left(1.91\times 10^{-2}\right)$-	$6.9527\times 10^{-1}\left(9.39\times 10^{-3}\right)$-	$8.5718\times 10^{-1}\left(9.25\times 10^{-3}\right)$
WFG6	$4.5343\times 10^{-1}\left(8.19\times 10^{-3}\right)$-	$4.7760\times 10^{-1}\left(5.86\times 10^{-3}\right)$-	$4.8313\times 10^{-1}\left(1.23\times 10^{-2}\right)$-	$4.8158\times 10^{-1}\left(6.40\times 10^{-3}\right)$-	$5.0356\times 10^{-1}$(6.67 × 10^−3^)+	$5.0093\times 10^{-1}\left(8.62\times 10^{-3}\right)$+	$4.9667\times 10^{-1}\left(5.46\times 10^{-3}\right)$
	$7.0486\times 10^{-1}\left(5.50\times 10^{-3}\right)$-	$6.8507\times 10^{-1}\left(6.78\times 10^{-3}\right)$-	$5.7081\times 10^{-1}\left(3.50\times 10^{-2}\right)$-	$6.5290\times 10^{-1}\left(7.68\times 10^{-3}\right)$-	$6.6223\times 10^{-1}\left(1.05\times 10^{-2}\right)$-	$6.9074\times 10^{-1}\left(9.37\times 10^{-3}\right)$-	$7.1423\times 10^{-1}$(7.94 × 10^−3^)
	$7.9217\times 10^{-1}\left(1.37\times 10^{-2}\right)$=	$7.4444\times 10^{-1}\left(1.56\times 10^{-2}\right)$-	$7.3104\times 10^{-1}\left(1.69\times 10^{-2}\right)$-	$6.4452\times 10^{-1}\left(3.14\times 10^{-2}\right)$-	$6.6074\times 10^{-1}\left(2.54\times 10^{-2}\right)$-	$6.7667\times 10^{-1}\left(1.65\times 10^{-2}\right)$-	$7.9859\times 10^{-1}$(1.12 × 10^−2^)
	$8.3666\times 10^{-1}\left(9.96\times 10^{-3}\right)$=	$7.9586\times 10^{-1}(1.48\times 10^{-2}$)-	$8.0707\times 10^{-1}\left(2.31\times 10^{-2}\right)$-	$6.2345\times 10^{-1}\left(4.07\times 10^{-2}\right)$-	$7.3322\times 10^{-1}\left(1.06\times 10^{-2}\right)$-	$6.6729\times 10^{-1}\left(1.75\times 10^{-2}\right)$-	$8.4062\times 10^{-1}$(1.07 × 10^−2^)
WFG7	$4.8073\times 10^{-1}\left(1.25\times 10^{-2}\right)$-	$5.1659\times 10^{-1}\left(3.71\times 10^{-3}\right)$-	$5.1847\times 10^{-1}\left(1.05\times 10^{-2}\right)$-	$5.1885\times 10^{-1}\left(4.28\times 10^{-3}\right)$-	$5.4118\times 10^{-1}$(2.72 × 10^−3^)+	$5.3606\times 10^{-1}\left(2.94\times 10^{-3}\right)$+	$5.3347\times 10^{-1}\left(2.74\times 10^{-3}\right)$
	$7.5540\times 10^{-1}\left(1.02\times 10^{-2}\right)$-	$7.4360\times 10^{-1}\left(6.97\times 10^{-3}\right)$-	$6.4797\times 10^{-1}\left(3.04\times 10^{-2}\right)$-	$7.0042\times 10^{-1}\left(1.19\times 10^{-2}\right)$-	$7.1973\times 10^{-1}\left(9.46\times 10^{-3}\right)$-	$7.3179\times 10^{-1}\left(7.72\times 10^{-3}\right)$-	$7.6633\times 10^{-1}$(3.32 × 10^−3^)
	$8.4556\times 10^{-1}\left(1.33\times 10^{-2}\right)$=	$8.0092\times 10^{-1}\left(9.39\times 10^{-3}\right)$-	$7.8178\times 10^{-1}\left(1.49\times 10^{-2}\right)$-	$7.1441\times 10^{-1}\left(2.46\times 10^{-2}\right)$-	$7.1694\times 10^{-1}\left(1.99\times 10^{-2}\right)$-	$6.8285\times 10^{-1}\left(2.53\times 10^{-2}\right)$-	$8.4933\times 10^{-1}$(9.73 × 10^−3^)
	$9.0335\times 10^{-1}$(3.51 × 10^−3^)+	$8.6441\times 10^{-1}\left(1.47\times 10^{-2}\right)$-	$8.5810\times 10^{-1}\left(3.12\times 10^{-2}\right)$-	$7.1347\times 10^{-1}\left(2.66\times 10^{-2}\right)$-	$8.0155\times 10^{-1}\left(2.78\times 10^{-2}\right)$-	$6.7013\times 10^{-1}\left(2.98\times 10^{-2}\right)$-	$8.9970\times 10^{-1}\left(6.79\times 10^{-3}\right)$
WFG8	$4.4265\times 10^{-1}\left(1.04\times 10^{-2}\right)$-	$4.5792\times 10^{-1}\left(4.13\times 10^{-3}\right)$-	$4.6680\times 10^{-1}\left(7.19\times 10^{-3}\right)$-	$4.6688\times 10^{-1}\left(5.87\times 10^{-3}\right)$-	$4.8214\times 10^{-1}\left(4.34\times 10^{-3}\right)$=	$4.8575\times 10^{-1}$(3.66 × 10^−3^)+	$4.8218\times 10^{-1}\left(3.67\times 10^{-3}\right)$
	$6.7930\times 10^{-1}\left(6.11\times 10^{-3}\right)$-	$6.4398\times 10^{-1}\left(7.12\times 10^{-3}\right)$-	$4.6913\times 10^{-1}\left(2.78\times 10^{-2}\right)$-	$6.2726\times 10^{-1}\left(7.28\times 10^{-3}\right)$-	$6.2434\times 10^{-1}\left(1.70\times 10^{-2}\right)$-	$6.6749\times 10^{-1}\left(5.97\times 10^{-3}\right)$-	$6.8695\times 10^{-1}$(4.60 × 10^−3^)
	$7.0930\times 10^{-1}\left(1.09\times 10^{-2}\right)$-	$6.9371\times 10^{-1}\left(3.57\times 10^{-2}\right)$-	$6.6825\times 10^{-1}\left(2.25\times 10^{-2}\right)$-	$5.3986\times 10^{-1}\left(4.05\times 10^{-2}\right)$-	$5.9331\times 10^{-1}\left(2.37\times 10^{-2}\right)$-	$5.7073\times 10^{-1}\left(1.58\times 10^{-2}\right)$-	$7.2028\times 10^{-1}$(1.47 × 10^−2^)
	$7.4222\times 10^{-1}\left(1.48\times 10^{-1}\right)$-	$8.1011\times 10^{-1}$(3.32 × 10^−2^)+	$7.7891\times 10^{-1}\left(1.18\times 10^{-2}\right)$-	$4.9857\times 10^{-1}\left(6.28\times 10^{-2}\right)$-	$6.6215\times 10^{-1}\left(2.80\times 10^{-2}\right)$-	$5.3859\times 10^{-1}\left(1.72\times 10^{-2}\right)$-	$7.9391\times 10^{-1}\left(1.85\times 10^{-2}\right)$
WFG9	$4.5502\times 10^{-1}\left(1.19\times 10^{-2}\right)$-	$4.8205\times 10^{-1}\left(1.02\times 10^{-2}\right)$-	$4.7668\times 10^{-1}\left(1.60\times 10^{-2}\right)$-	$4.7424\times 10^{-1}\left(1.34\times 10^{-2}\right)$-	$4.9522\times 10^{-1}$(2.24 × 10^−2^)+	$4.8067\times 10^{-1}\left(1.47\times 10^{-2}\right)$-	$4.9076\times 10^{-1}\left(1.45\times 10^{-2}\right)$
	$6.8052\times 10^{-1}\left(1.28\times 10^{-2}\right)$=	$6.7580\times 10^{-1}\left(1.19\times 10^{-2}\right)$-	$6.0375\times 10^{-1}\left(4.07\times 10^{-2}\right)$-	$6.2373\times 10^{-1}\left(1.57\times 10^{-2}\right)$-	$6.8657\times 10^{-1}$(8.38 × 10^−3^)=	$6.2175\times 10^{-1}\left(1.87\times 10^{-2}\right)$-	$6.8623\times 10^{-1}\left(1.70\times 10^{-2}\right)$
	$7.4097\times 10^{-1}\left(2.76\times 10^{-2}\right)$=	$7.0294\times 10^{-1}\left(2.40\times 10^{-2}\right)$-	$6.6238\times 10^{-1}\left(4.6\times 10^{-2}\right)$-	$6.0242\times 10^{-1}\left(2.83\times 10^{-2}\right)$-	$6.9792\times 10^{-1}\left(2.40\times 10^{-2}\right)$-	$5.1020\times 10^{-1}\left(3.65\times 10^{-2}\right)$-	$7.4672\times 10^{-1}$(2.74 × 10^−2^)
	$7.9962\times 10^{-1}$(1.88 × 10^−2^)=	$7.8538\times 10^{-1}\left(3.74\times 10^{-2}\right)$=	$7.5141\times 10^{-1}\left(3.08\times 10^{-2}\right)$-	$6.5714\times 10^{-1}\left(2.78\times 10^{-2}\right)$-	$7.6339\times 10^{-1}\left(1.75\times 10^{-2}\right)$-	$5.2967\times 10^{-1}\left(2.32\times 10^{-2}\right)$-	$7.9674\times 10^{-1}\left(2.29\times 10^{-2}\right)$
+/-/=	15/32/17	8/31/25	29/28/7	16/42/6	19/34/11	13/37/14	

## Data Availability

Data is contained within the article.
